# New adaptive EWMA CV control chart with application to the sintering process

**DOI:** 10.1038/s41598-024-62316-4

**Published:** 2024-05-21

**Authors:** Sadaf Ayesha, Asma Arshad, Olayan Albalawi, Aiedh Mrisi Alharthi, Muhammad Hanif, Uzma Yasmeen, Muhammad Nabi

**Affiliations:** 1 Department of Statistics, National College of Business Administration and Economics, Lahore, Pakistan; 2 Department of Statistics, Faculty of Science, University of Tabuk, Tabuk, Saudi Arabia; 3 Department of Mathematics, Turabah University College, Taif University, Taif, Saudi Arabia; 4 Brock University, St. Catharines, Canada; 5 Khost Mechanics Institute, Khost, Afghanistan

**Keywords:** Statistical process control, Adaptive control charts, EWMA, Coefficient of variation, Average run length, Standard deviation run length, Engineering, Materials science

## Abstract

This research presents a new adaptive exponentially weighted moving average control chart, known as the coefficient of variation (CV) EWMA statistic to study the relative process variability. The production process CV monitoring is a long-term process observation with an unstable mean. Therefore, a new modified adaptive exponentially weighted moving average (AAEWMA) CV monitoring chart using a novel function hereafter referred to as the "AAEWMA CV" monitoring control chart. the novelty of the suggested AAEWMA CV chart statistic is to identify the infrequent process CV changes. A continuous function is suggested to be used to adapt the plotting statistic smoothing constant value as per the process estimated shift size that arises in the CV parametric values. The Monte Carlo simulation method is used to compute the run-length values, which are used to analyze efficiency. The existing AEWMA CV chart is less effective than the proposed AAEWMA CV chart. An industrial data example is used to examine the strength of the proposed AAEWMA CV chart and to clarify the implementation specifics which is provided in the example section. The results strongly recommend the implementation of the proposed AAEWMA CV control chart.

## Introduction

The field of statistical process control (SPC) offers straightforwardly effective instruments for industrial unit monitoring. The most encouraging aspect is the persistent work of the researchers, who offer tools appropriate for the expanding needs of industry Mondal et al.^1^. Shewhart^2^ has made the first effort and is the result of the initial attempt at Bell Laboratories. Shewhart^2^ created a monitoring chart with two control boundaries on it as upper control limit and the lower control limit. Shewhart charts with a time frame are effective in identifying only significant process alterations, although they were designed to identify changes in the process mean, variance, and standard deviation as well. Control charts with memory type function statistics are recommended for monitoring production units that are susceptible to even slight shifts. There are two basic types of memory type control charts: the exponentially weighted moving average (EWMA) control chart by Roberts^3^ and the cumulative sum (CUSUM) control chart provided by Page^4^. Additionally, several altered variants of memory charts are created by SPC researchers to quickly manage minor to moderate process shifts. Amongst them, the work of a few recent citations are as follows: Sanusi et al.^5^ have given the concept of Max EWMA, Alevizokos et al.^6^ proposed a triple EWMA memory control chart, Noor-ul-Amin et al.^7^ suggested a max EWMA control chart using measurement error technique for monitoring auxiliary information based data set, Alduais et al.^8^ have suggested EWMA using engineering based applications using the Rayleigh distribution. To identify changes rapidly, the adaptation of the constant parameter is suggested in both types of memory monitoring charts while monitoring mean, variance, or standard deviation (SD), etc., in a process. However, there are situations when the mean value varies from process unit to process unit calculations at specified parameters. In that instance, it is advised to keep an eye/monitor the constant proportionality between the mean and the SD as the process unit's coefficient of variation (CV).

The ratio of process standard deviation to mean, or variance, whereas this ratio is a linear function of mean to give a relative measure of process variability that is examined using the CV control chart. it is important to keep in mind that all monitoring is sample-based which is the uniqueness of an adaptive control chart design. The design of control charts is typically made to monitor variations or the process means to identify unfavorable changes (the shifts). In conventional situations, one process parameter, such as mean, standard deviation, variance, range, etc., is monitored while the other stays constant for a variable under investigation. However, if the mean is not constant over time, then using a mean or variance monitoring control chart is not practical. So, if it would be viewed that sample variance at some time with some linear relation to a function of mean and shows some proportionality Kang et al.^9^. Then it is better to apply a CV monitoring chart for identifying changes in both the mean and variance. Additionally, using a CV control chart to track the variability of emerging process alterations is a smart practice. Variance is defined as the linear function of the mean; hence, the CV monitoring chart is recommended to identify changes in both the mean and variance. To further track the variability of the emerging process shifts, the CV control chart is a useful tool. Furthermore, CV charts are not used unless the CV's constant ratio, value, or domain is expected to remain unchanged. This constant ratio situation is explained by a few instances from the two main fields: die-casting hot chamber weight monitoring by Castagliola et al.^10^ and chemistry of patient's blood analysis in a specific disease by Kang et al.^9^. Some recent workings on CV control charts proved efficient in SPC literature: Abbasi^11^ has presented a CV control chart by using some auxiliary variables as some added information, Noor-ul-Amin et al.^12^ have given CV EWMA control chart by using some lognormal transformation by using the ranked set sampling procedures as a variant sampling techniques, and Tran et al.^13^ have suggested a one-sided run rules-based control chart in the presence of some measurement errors.

As the Shewhart^14^ charts provided the foundation for SPC, the original SPC pioneer, Kang et al.^9^ is a pioneer of the Shewhart CV chart. SPC researchers are forced by the creation of Shewhart charts to create more effective monitoring designs that are adaptable to the changes in running production requirements. The CV monitoring charts are a significant advancement, and Kang et al.^9^ research demonstrated their effectiveness in identifying significant process CV shifts. To more effectively detect minor to moderate CV shifts, Hong et al.^15^ recommend one additional sensitive modification: an EWMA CV chart. Castagliola et al.^16^'s proposal, which included two one-sided EWMA CV squared charts, proved to be more effective in quickly identifying CV shifts. An EWMA CV chart was also provided by Zhang et al.^17^, who judged it to be superior to Castagliola et al.^16^'s. In terms of enhanced sensitivity, Calzada and Scariano^18^ integrated a CV synthetic chart that had a better form than the counterparts EWMA CV^2^ and EWMA CV charts. The run rules strategy is suggested by Castagliola et al.^19^ to improve the design of the CV chart. Yeong et al.^20^ made changes to the CV chart by observing measurement errors in the discussion as a main part of the design. A run sum CV control chart was introduced by Teoh et al.^21^, who found it to be effective. A variable sample size EWMA CV chart was recently proposed by Muhammad et al.^22^, while a variable parameter CV monitoring chart was recently proposed by Yeong et al.^23^. In the context of quality control, Noor-ul-Amin et al.^24^ introduced a concurrent process monitoring approach for both the process mean and CV in the presence of auxiliary and non-auxiliary data. Similarly, Riaz and Noor-ul-Amin^25^ presented a simultaneous method for monitoring the process mean and CV when ranked set sampling is employed.

In the realm of statistical quality control, Roberts^3^ initially introduced the EWMA statistic as a weighting scheme. This scheme assigns weights to previously computed units and incorporates a smoothing constant (SC) value for the presently computed unit. By maintaining a record and utilizing previous observation results, the corresponding SC allocates the remaining weight among previous observations, creating what is termed a memory control chart. Due to this property, EWMA charts are sensitive to slight to moderate shifts. Specifically, the SC value is assigned to the chart to implement it as a known shift size practice, assuming that process shifts are known. In practical terms, it seems unreasonable to use a chart without shift estimation or a meaningful method to determine the purported shift size. The adaptive control chart emerges as a logical approach in the SPC literature for estimating the developing process shift size. The SC value is then adjusted based on the computed shift. With the SC value appropriately shaped, this approach, known as the adaptive EWMA (AEWMA) control chart in SPC literature by Capizzi and Masarotto^26^ has proven effective in detecting shifts of any size. In comparison, the Shewhart, EWMA, and Shewhart EWMA classical control charts are less effective at detecting changing patterns in parametric values than the AEWMA chart, thanks to this adaptive approach. Haq and Khoo^27^ proposed a novel approach to AEWMA methodology which involves the selection of the SC value using a step function dependent on the shift magnitude that is determined by an unbiased estimator. Using the Bayesian technique under various loss functions, Noor-ul-Amin and Noor^28^ presented an AEWMA mean control chart and they found it to be more efficient than the charts already in use.

In the context of SPC, this study introduces a novel approach for effectively monitoring the process CV through the development of an AAEWMA CV control chart by using a novel function approach. The design of the proposed chart aims to enhance sensitivity by incorporating the CV as a monitoring tool within the adaptive framework recommended by Sarwar and Noor-ul-Amin^29^, Arshad et al.^30^, Noor et al.^31^, and others. The primary objective is to determine the SC value by predicting the shift size of the process CV parametric value. The incorporation of the function which was given by Sarwar and Noor-ul-Amin^29^ has been identified to improve the computations within the architecture of the proposed AAEWMA CV chart. Run-length (RL) values are a tool to assess the performance of any chart so, the suggested AAEWMA CV chart undergoes evaluation based on these RL values. Monte Carlo (MC) simulation methods are utilized for RL value calculations involving 100,000 iterations for each simulation run repeated 100,000 times. Tables 1, 2, 3 present the computed RL findings and offer an assessment of the proficiency levels compared to existing adaptive charts. To provide a clearer interpretation of the comparative excellence of the result, the average and standard deviation of the RL values are presented as average RL (ARL) and standard deviation of RL (SDRL). Montgomery^32^ recommends that the computed ARL and SDRL should be lower than their counterparts to demonstrate the sensitivity of the proposed AAEWMA CV chart concept. The RL of any control chart is recorded when the plotted statistic crosses the limit or in-control threshold of the proposed AAEWMA CV chart.

**Table 1:** The proposed AAEWMA CV chart two-sided RL profile for ${ARL}_{0}\cong 370$.

${\varvec{\delta}}$	AAEWMA CV chart two-sided increased/decreased shift with $\psi$ $=0.1$ and $\mathfrak{h}=0.3379$												
		${\gamma }_{0}=0.10$				${\gamma }_{0}=0.15$				${\gamma }_{0}=0.20$			
		$n=3$	$n=5$	$n=8$	$n=10$	$n=3$	$n=5$	$n=8$	$n=10$	$n=3$	$n=5$	$n=8$	$n=10$
0.25	${{\varvec{A}}{\varvec{R}}{\varvec{L}}}_{1}$	2.47	1.60	1.05	1.00	2.48	1.60	1.05	1.00	2.48	1.61	1.05	1.00
	${SDRL}_{1}$	0.75	0.50	0.22	0.05	0.75	0.50	0.22	0.06	0.75	0.50	0.23	0.06
0.50	${{\varvec{A}}{\varvec{R}}{\varvec{L}}}_{1}$	5.92	3.13	2.09	1.80	5.92	3.14	2.11	1.81	5.98	3.17	2.12	1.82
	${SDRL}_{1}$	3.15	1.30	0.70	0.59	3.13	1.31	0.71	0.59	3.21	1.33	0.72	0.60
0.55	${{\varvec{A}}{\varvec{R}}{\varvec{L}}}_{1}$	7.38	3.78	2.45	2.07	7.44	3.79	2.47	2.10	7.49	3.85	2.50	2.10
	${SDRL}_{1}$	4.32	1.77	0.93	0.73	4.36	1.79	0.95	0.74	4.38	1.83	0.95	0.75
0.60	${{\varvec{A}}{\varvec{R}}{\varvec{L}}}_{1}$	9.52	4.72	2.95	2.48	9.53	4.71	2.98	2.49	9.60	4.77	3.00	2.51
	${SDRL}_{1}$	6.02	2.48	1.28	0.98	6.04	2.46	1.29	0.98	6.12	2.51	1.31	1.01
0.65	${{\varvec{A}}{\varvec{R}}{\varvec{L}}}_{1}$	12.64	6.10	3.69	3.03	12.56	6.11	3.72	3.05	12.66	6.18	3.76	3.11
	${SDRL}_{1}$	8.51	3.56	1.81	1.36	8.53	3.57	1.84	1.38	8.61	3.61	1.87	1.42
0.70	${{\varvec{A}}{\varvec{R}}{\varvec{L}}}_{1}$	17.06	8.19	4.86	3.91	17.25	8.29	4.87	3.97	17.22	8.43	4.94	4.02
	${SDRL}_{1}$	12.14	5.32	2.71	2.02	12.26	5.34	2.73	2.08	12.19	5.48	2.80	2.08
0.75	${{\varvec{A}}{\varvec{R}}{\varvec{L}}}_{1}$	24.22	11.58	6.75	5.37	24.30	11.76	6.81	5.42	24.60	11.90	6.92	5.53
	${SDRL}_{1}$	17.86	8.12	4.25	3.21	17.80	8.26	4.34	3.22	17.98	8.32	4.45	3.34
0.80	${{\varvec{A}}{\varvec{R}}{\varvec{L}}}_{1}$	35.63	17.76	10.18	8.05	35.45	18.11	10.31	8.16	35.89	18.27	10.55	8.30
	${SDRL}_{1}$	26.62	13.14	7.17	5.44	26.69	13.43	7.28	5.53	26.90	13.55	7.49	5.62
0.85	${{\varvec{A}}{\varvec{R}}{\varvec{L}}}_{1}$	55.29	29.69	17.37	13.82	55.97	29.82	17.74	13.89	56.02	30.35	18.07	14.20
	${SDRL}_{1}$	42.02	22.64	13.21	10.32	42.00	22.77	13.49	10.39	42.68	23.19	13.76	10.66
0.90	${{\varvec{A}}{\varvec{R}}{\varvec{L}}}_{1}$	95.17	55.74	34.79	28.05	95.36	55.53	35.14	28.30	95.19	56.65	35.82	29.07
	${SDRL}_{1}$	76.32	43.15	27.34	22.19	76.41	43.45	27.63	22.22	76.84	44.58	28.24	22.78
0.95	${{\varvec{A}}{\varvec{R}}{\varvec{L}}}_{1}$	202.74	133.80	91.53	77.06	203.53	133.90	92.45	77.92	204.16	136.25	94.96	79.19
	${SDRL}_{1}$	190.17	118.00	76.92	64.01	191.77	118.11	77.36	64.65	193.93	120.00	79.50	65.37
1.00	${{\varvec{A}}{\varvec{R}}{\varvec{L}}}_{0}$	370.91	370.18	370.24	370.59	370.40	370.85	373.32	369.97	372.69	368.31	372.65	373.59
	${SDRL}_{0}$	407.30	407.74	408.20	410.12	405.43	403.42	408.18	403.65	408.87	405.73	410.58	409.42
1.05	${{\varvec{A}}{\varvec{R}}{\varvec{L}}}_{1}$	168.25	116.34	82.59	70.49	167.83	117.41	83.57	71.22	170.48	117.88	85.25	73.45
	${SDRL}_{1}$	171.35	111.94	74.85	63.44	170.13	111.55	75.59	64.23	172.22	112.63	77.45	65.41
1.10	${{\varvec{A}}{\varvec{R}}{\varvec{L}}}_{1}$	78.24	48.19	32.26	26.36	79.69	48.63	32.69	26.67	79.88	50.11	32.95	27.21
	${SDRL}_{1}$	72.95	43.56	28.44	22.96	75.12	43.56	28.67	23.42	75.02	44.77	28.99	23.69
1.20	${{\varvec{A}}{\varvec{R}}{\varvec{L}}}_{1}$	30.76	17.15	10.72	8.58	30.97	17.53	10.89	8.85	31.52	17.91	11.23	9.05
	${SDRL}_{1}$	28.18	15.32	9.14	7.09	28.54	15.63	9.30	7.31	28.95	16.06	9.56	7.49
1.30	${{\varvec{A}}{\varvec{R}}{\varvec{L}}}_{1}$	16.63	9.17	5.77	4.71	16.82	9.32	5.86	4.82	17.17	9.53	6.01	4.95
	${SDRL}_{1}$	15.41	7.89	4.51	3.47	15.65	8.06	4.58	3.57	15.92	8.34	4.73	3.68
1.40	${{\varvec{A}}{\varvec{R}}{\varvec{L}}}_{1}$	10.86	5.97	3.84	3.24	10.95	6.14	3.93	3.30	11.10	6.26	4.06	3.37
	${SDRL}_{1}$	9.90	4.81	2.72	2.14	10.00	5.01	2.80	2.18	10.06	5.14	2.93	2.27
1.50	${{\varvec{A}}{\varvec{R}}{\varvec{L}}}_{1}$	7.83	4.40	2.91	2.48	7.89	4.47	2.98	2.53	8.05	4.61	3.06	2.58
	${SDRL}_{1}$	6.95	3.36	1.91	1.49	7.02	3.42	1.96	1.54	7.13	3.59	2.02	1.58
1.60	${{\varvec{A}}{\varvec{R}}{\varvec{L}}}_{1}$	6.06	3.48	2.37	2.05	6.16	3.56	2.43	2.08	6.24	3.64	2.50	2.14
	${SDRL}_{1}$	5.20	2.52	1.43	1.15	5.34	2.56	1.49	1.17	5.39	2.68	1.54	1.23
1.70	${{\varvec{A}}{\varvec{R}}{\varvec{L}}}_{1}$	4.94	2.91	2.04	1.77	4.99	2.96	2.08	1.79	5.12	3.06	2.14	1.85
	${SDRL}_{1}$	4.11	1.98	1.15	0.92	4.13	2.04	1.19	0.95	4.28	2.10	1.24	0.99
1.80	${{\varvec{A}}{\varvec{R}}{\varvec{L}}}_{1}$	4.18	2.52	1.81	1.57	4.25	2.57	1.83	1.60	4.33	2.64	1.88	1.64
	${SDRL}_{1}$	3.34	1.65	0.98	0.78	3.47	1.68	0.99	0.78	3.53	1.78	1.02	0.81
1.90	${{\varvec{A}}{\varvec{R}}{\varvec{L}}}_{1}$	3.65	2.25	1.62	1.44	3.69	2.30	1.65	1.46	3.78	2.34	1.70	1.49
	${SDRL}_{1}$	2.86	1.40	0.82	0.66	2.89	1.43	0.85	0.68	2.96	1.49	0.89	0.71
2.00	${{\varvec{A}}{\varvec{R}}{\varvec{L}}}_{1}$	3.22	2.03	1.50	1.33	3.30	2.07	1.53	1.36	3.34	2.13	1.57	1.39
	${SDRL}_{1}$	2.44	1.22	0.72	0.57	2.51	1.26	0.75	0.59	2.56	1.30	0.78	0.62
2.25	${{\varvec{A}}{\varvec{R}}{\varvec{L}}}_{1}$	2.20	1.48	1.18	1.10	2.23	1.52	1.20	1.11	2.29	1.56	1.22	1.13
	${SDRL}_{1}$	1.48	0.73	0.42	0.31	1.49	0.78	0.45	0.33	1.56	0.80	0.47	0.36
3.00	${{\varvec{A}}{\varvec{R}}{\varvec{L}}}_{1}$	1.77	1.27	1.07	1.03	1.79	1.29	1.09	1.04	1.85	1.33	1.11	1.05
	${SDRL}_{1}$	1.06	0.53	0.27	0.18	1.10	0.55	0.29	0.20	1.14	0.60	0.33	0.23

**Table 2:** The proposed AAEWMA CV chart one-sided increase CV shift RL profile for ${ARL}_{0}\cong 370$.

${\varvec{\delta}}$	Proposed AAEWMA CV chart one-sided increase CV shift RL profile with $\psi$ $=0.1$ and $\mathfrak{h}=0.1946$												
		${\gamma }_{0}=0.10$				${\gamma }_{0}=0.15$				${\gamma }_{0}=0.20$			
		$n=3$	$n=5$	$n=8$	$n=3$	$n=5$	$n=8$	$n=3$	$n=5$	$n=8$	$n=3$	$n=5$	$n=8$
1.00	${{\varvec{A}}{\varvec{R}}{\varvec{L}}}_{0}$	370.74	370.34	370.93	370.47	370.71	370.78	370.17	370.95	370.92	370.71	370.82	370.12
	${SDRL}_{0}$	470.55	467.07	470.32	470.15	466.24	471.41	466.35	464.30	467.89	461.82	468.22	464.40
1.05	${{\varvec{A}}{\varvec{R}}{\varvec{L}}}_{1}$	100.13	67.18	48.71	41.28	101.26	68.61	49.10	41.92	101.28	69.14	50.44	42.74
	${SDRL}_{1}$	117.48	77.00	54.53	45.94	118.49	78.28	54.80	46.39	119.95	79.24	56.57	47.71
1.10	${{\varvec{A}}{\varvec{R}}{\varvec{L}}}_{1}$	47.27	29.08	18.85	15.84	47.53	29.77	19.50	16.00	48.03	29.88	19.84	16.34
	${SDRL}_{1}$	54.18	32.70	20.75	17.43	54.54	33.78	21.69	17.58	55.10	33.61	22.07	17.86
1.20	${{\varvec{A}}{\varvec{R}}{\varvec{L}}}_{1}$	19.24	10.70	6.72	5.59	19.42	10.92	6.90	5.65	19.43	11.13	7.10	5.85
	${SDRL}_{1}$	22.08	11.97	6.91	5.53	22.37	11.96	7.21	5.61	22.45	12.35	7.40	5.83
1.30	${{\varvec{A}}{\varvec{R}}{\varvec{L}}}_{1}$	10.73	5.98	3.82	3.16	10.87	6.03	3.90	3.22	10.97	6.22	4.01	3.32
	${SDRL}_{1}$	12.18	6.22	3.49	2.70	12.28	6.27	3.57	2.77	12.33	6.53	3.75	2.89
1.40	${{\varvec{A}}{\varvec{R}}{\varvec{L}}}_{1}$	7.12	4.01	2.67	2.27	7.14	4.10	2.71	2.29	7.40	4.16	2.79	2.36
	${SDRL}_{1}$	7.83	3.86	2.14	1.69	7.82	3.98	2.21	1.71	8.06	4.07	2.30	1.78
1.50	${{\varvec{A}}{\varvec{R}}{\varvec{L}}}_{1}$	5.28	3.06	2.08	1.79	5.38	3.10	2.12	1.83	5.37	3.18	2.18	1.88
	${SDRL}_{1}$	5.57	2.73	1.51	1.16	5.71	2.77	1.54	1.19	5.72	2.82	1.62	1.26
1.60	${{\varvec{A}}{\varvec{R}}{\varvec{L}}}_{1}$	4.18	2.49	1.76	1.54	4.23	2.53	1.79	1.57	4.31	2.58	1.82	1.61
	${SDRL}_{1}$	4.26	2.03	1.14	0.87	4.36	2.09	1.16	0.90	4.36	2.13	1.21	0.96
1.70	${{\varvec{A}}{\varvec{R}}{\varvec{L}}}_{1}$	3.51	2.12	1.55	1.38	3.53	2.17	1.58	1.39	3.58	2.21	1.61	1.43
	${SDRL}_{1}$	3.42	1.59	0.90	0.68	3.40	1.65	0.93	0.71	3.43	1.68	0.96	0.74
1.80	${{\varvec{A}}{\varvec{R}}{\varvec{L}}}_{1}$	2.99	1.89	1.40	1.27	3.02	1.91	1.43	1.28	3.08	1.95	1.45	1.31
	${SDRL}_{1}$	2.75	1.31	0.73	0.55	2.79	1.36	0.76	0.58	2.84	1.40	0.78	0.61
1.90	${{\varvec{A}}{\varvec{R}}{\varvec{L}}}_{1}$	2.67	1.70	1.31	1.20	2.70	1.72	1.33	1.21	2.73	1.78	1.36	1.23
	${SDRL}_{1}$	2.36	1.10	0.62	0.47	2.40	1.13	0.63	0.49	2.42	1.19	0.67	0.51
2.00	${{\varvec{A}}{\varvec{R}}{\varvec{L}}}_{1}$	2.38	1.58	1.24	1.14	2.45	1.60	1.26	1.16	2.48	1.64	1.28	1.17
	${SDRL}_{1}$	1.98	0.95	0.54	0.39	2.09	0.99	0.55	0.41	2.11	1.03	0.58	0.44
2.25	${{\varvec{A}}{\varvec{R}}{\varvec{L}}}_{1}$	1.74	1.25	1.08	1.04	1.75	1.27	1.09	1.04	1.79	1.30	1.10	1.05
	$SDRL_{1}$	1.19	0.56	0.28	0.19	1.21	0.58	0.30	0.20	1.27	0.62	0.32	0.22
3.00	${\varvec{ARL}}_{1}$	1.46	1.14	1.03	1.01	1.49	1.15	1.03	1.01	1.51	1.17	1.04	1.02
	$SDRL_{1}$	0.85	0.39	0.17	0.10	0.88	0.41	0.18	0.12	0.91	0.44	0.21	0.13

**Table 3:** The proposed AAEWMA CV chart one-sided decrease CV shift RL profile for $ARL_{0} \cong 370$.

${\varvec{\delta}}$	Proposed AAEWMA CV chart one-sided decrease CV shift RL profile with $\psi$ $= 0.1$ and ${\mathbf{\mathfrak{h}}} = 0.1946$												
		$\gamma_{0} = 0.10$				$\gamma_{0} = 0.15$				$\gamma_{0} = 0.20$			
		$n = 3$	$n = 5$	$n = 8$	$n = 3$	$n = 5$	$n = 8$	$n = 3$	$n = 5$	$n = 8$	$n = 3$	$n = 5$	$n = 8$
1.00	${\varvec{ARL}}_{0}$	370.53	370.42	370.26	370.85	370.32	370.10	370.51	368.25	370.12	370.57	370.99	369.66
	$SDRL_{0}$	464.62	466.86	462.58	465.88	460.91	461.59	469.00	463.78	467.90	466.88	465.03	462.35
0.95	${\varvec{ARL}}_{1}$	108.31	71.56	50.48	42.50	107.61	71.71	50.77	43.21	108.30	72.67	51.78	43.79
	$SDRL_{1}$	119.88	77.94	53.43	45.07	119.35	78.89	54.04	45.51	121.10	79.10	55.06	46.70
0.90	${\varvec{ARL}}_{1}$	51.08	30.29	19.13	15.62	51.42	30.05	19.54	15.89	51.25	30.92	19.90	15.96
	$SDRL_{1}$	52.59	30.60	19.04	15.54	53.02	30.70	19.65	15.72	53.07	31.34	19.92	16.01
0.85	${\varvec{ARL}}_{1}$	29.64	16.34	9.94	7.95	29.57	16.31	9.99	8.14	29.60	16.68	10.21	8.22
	$SDRL_{1}$	29.37	15.75	9.23	7.16	29.31	15.87	9.36	7.45	29.31	16.29	9.56	7.50
0.80	${\varvec{ARL}}_{1}$	19.12	10.03	6.05	4.84	19.18	10.06	6.14	4.92	19.24	10.21	6.17	4.98
	$SDRL_{1}$	18.18	9.20	5.10	3.86	18.16	9.18	5.18	3.93	18.37	9.29	5.19	4.03
0.75	${\varvec{ARL}}_{1}$	13.17	6.78	4.11	3.33	13.28	6.77	4.15	3.41	13.36	6.92	4.22	3.43
	$SDRL_{1}$	12.07	5.63	3.03	2.30	12.23	5.69	3.10	2.38	12.32	5.89	3.17	2.40
0.70	${\varvec{ARL}}_{1}$	9.51	4.88	3.03	2.51	9.61	4.90	3.08	2.52	9.70	5.00	3.10	2.55
	$SDRL_{1}$	8.24	3.71	1.98	1.51	8.33	3.77	2.03	1.53	8.42	3.84	2.03	1.55
0.65	${\varvec{ARL}}_{1}$	7.11	3.68	2.35	1.99	7.18	3.74	2.38	2.00	7.18	3.77	2.41	2.04
	$SDRL_{1}$	5.68	2.51	1.35	1.05	5.77	2.57	1.37	1.06	5.81	2.58	1.40	1.08
0.60	${\varvec{ARL}}_{1}$	5.52	2.95	1.94	1.64	5.56	2.96	1.93	1.65	5.60	2.99	1.97	1.67
	$SDRL_{1}$	4.17	1.80	0.98	0.76	4.19	1.81	0.98	0.77	4.23	1.84	1.00	0.78
0.55	${\varvec{ARL}}_{1}$	4.43	2.40	1.62	1.39	4.44	2.41	1.63	1.41	4.44	2.42	1.64	1.41
	$SDRL_{1}$	3.05	1.30	0.73	0.57	3.06	1.30	0.73	0.59	3.04	1.32	0.74	0.59
0.50	${\varvec{ARL}}_{1}$	3.61	2.01	1.39	1.22	3.62	2.03	1.40	1.23	3.62	2.03	1.41	1.23
	$SDRL_{1}$	2.25	0.98	0.56	0.43	2.26	1.00	0.57	0.44	2.26	0.99	0.57	0.44
0.25	${\varvec{ARL}}_{1}$	1.61	1.05	1.00	1.00	1.61	1.05	1.00	1.00	1.62	1.05	1.00	1.00
	$SDRL_{1}$	0.61	0.22	0.01	0.00	0.61	0.22	0.01	0.00	0.61	0.23	0.01	0.00

The manuscript's other portions are arranged as follows: The construction of the suggested monitoring AAEWMA CV chart is explained in section "Proposed control chart". Section "Comparitive analysis" outlines the comparative study with the current charts to demonstrate the sensitivity of the current adaptive designs, while section "Performance Evaluation" discusses the computed RL results. In section "Real life example", an industrial dataset application is given. The respective limitations and the future recommendations concerning the proposed control chart are mentioned in sections "Limitations" and "Future recommendations". The study's conclusions are summarized in section "Conclusion", which also includes a brief discussion of potential avenues for future research to expand the topic under consideration. An illustrative example is also provided based on some hypothetical dataset and extensively provides the comparison picture with the existing counterpart in section "Illustrative Example".

## Proposed control chart

In this section, the design of the proposed "AAEWMA CV control chart," is provided as a novel function given by Sarwar and Noor-ul-Amin^29^, an AEWMA CV control chart that is suggested to keep an eye on the sporadic modifications to a normally distributed production CV. The variable of interest, *Y*, has a mean of ${\mu }_{Y}$ and a variance of ${\sigma }_{Y}^{2}$. Its variable, *Y*, is distributed normally as $Y\sim N\left({{\mu }_{Y},\sigma }_{Y}^{2}\right)$. When time $t \ge 1$, the production units $\left\{{Y}_{t}\right\}$ follows a normal distribution and is sequenced. The mean ${\mu }_{Y}$ and standard deviation ${\sigma }_{Y}$ are fixed to get the CV ${\gamma }_{{Y}_{t}}$ computed as ${\gamma }_{{Y}_{t}}=\frac{{\sigma }_{{Y}_{t}}}{{\mu }_{{Y}_{t}}},$ suggests that while the values of the parameters ${\sigma }_{{Y}_{t}}$ and ${\mu }_{{Y}_{t}}$ vary individually in each group by group and the ratio of the two parameters like ${\mu }_{Y}$ and ${\sigma }_{Y}$ be taken as ${\gamma }_{{Y}_{t}}$, the value of ${\gamma }_{{Y}_{t}}$ supposed to stay constant across time $t$ for an in control process.

Hence, the in-control process ${\gamma }_{{Y}_{t}}$ monitoring is computed as ${\gamma }_{{Y}_{t=0}}$ and using a sample with $n$ size that is drawn from simple random sampling. At time $t>{t}_{0}$ the sequence of $Y$ values is $\left\{{Y}_{t}\right\}$ as random units of observations $\left\{{Y}_{1t},{Y}_{2t},\dots ,{Y}_{nt}\right\}$; ${Y}_{it}$ is the sample’s ith observation for $i = 1,2, \ldots ,n$ at $t$ respectively. The sequenced CV values for the production $\left\{ {Y_{t} } \right\}$ are calculated using the corresponding sample means $\overline{{Y }_{t}}=\sum_{i=1}^{n}{Y}_{it}/n$ and the standard deviations ${S}_{t}=\sqrt{{\sum }_{i=1}^{n}{\left({Y}_{it}-\overline{{Y }_{t}}\right)}^{2}/(n-1)}$. As $\left\{{\widehat{\gamma }}_{t}=\frac{{S}_{t}}{\overline{{Y }_{t}}}\right\}$, is a respective sequence of random variables for the time domain $t>1,$ and the sample estimates ${\widehat{\gamma }}_{t}$ which are defined with a range of $(0,\infty )$, is the sequenced CV value. The non-central $t$-distribution for $(n-1)$ degrees of freedom is followed in this case by $\left\{\frac{\sqrt{n}}{{\widehat{\gamma }}_{t}}\right\}$ and the non-centrality parameter becomes $\frac{\sqrt{n}}{{\gamma }_{t}}.$ The noncentral $F$ distribution, in this case, is represented by $\frac{n}{{{\widehat{\gamma }}_{t}}^{2}}$ and the $(1, n-1)$ degrees of freedom with $\frac{n}{{\gamma }_{t}^{2}}$ is the non-centrality parameter, such as $\frac{n}{{{\widehat{\gamma }}_{t}}^{2}} \sim F( 1, n-1, \frac{n}{{\gamma }_{t}^{2}} )$. The statistic becomes normally distributed and the cumulative distribution function, as CDF transformation is characterized as ${F}_{F}$ for the respective non-central *F* distribution and it makes the statistic ${F}_{F}$ as a uniformly distributed random variable which is followed by; ${F}_{F}(\frac{n}{{{\widehat{\gamma }}_{t}}^{2}};1,n-1,\frac{n}{{\gamma }_{t}^{2}} ) \sim U( 0, 1)$. Additively, the CDF inverse transforms the respective uniform variable into a standard normal random variable as ${Y}_{t}^{*}={\Phi }^{-1}\left({F}_{F}\left(\frac{n}{{{\widehat{\gamma }}_{t}}^{2}};1,n-1,\frac{n}{{\gamma }_{t}^{2}} \right)\right) \sim U( 0, 1)$, followed by the standard normal distribution. Here, ${\Phi }^{-1}\left(.\right)$ is the respective inverse CDF of the standard normal distribution.

Now, the $Y_{t}^{*} \sim N\left( {0,1} \right)$, is the representation of the statistic $Y_{t}^{*}$ which followed the respective standard normal distribution after some transformations as discussed earlier. To construct a CV control chart, the RL values are proposed to be recorded at ${\gamma }_{t=0}$ while monitoring the process CV. The basic CV chart methodology assumes that the shift ${{\varvec{\delta}}}_{t}$ size at some specified time $t$ is ${{\varvec{\delta}}}_{t}=E({Y}_{t}^{*})$ with ${{\varvec{\delta}}}_{t}=0,$ an in-control process state for $t\le {t}_{0}$. But as the process faced some shift and gave an indication for the out-of-control process, the shift of the process is taken as ${{\varvec{\delta}}}_{t}\ne 0$ at $t>{t}_{0}$ and the computed CV shift state becomes ${\gamma }_{y=1}{\ne \gamma }_{y=0}$. However, as the process shifted from in-control to out-of-control, it caused a change in the variability of the process and the statistic ${Y}_{t}^{*}$. After normalizing the sequence of ${Y}_{t}^{*}$ values are taken as $\left\{{Y}_{t}^{*}\right\}$. The process computed CV value in the presence of some shift ${\varvec{\delta}}$ is determined with an unbiased estimator which was originally introduced by Jiang et al.^33^. The theory is to estimate the shift from the sample values drawn at the time $t$. So, the shift ${\varvec{\delta}}$ is estimated using the EWMA statistic, as follows:

1 $${\widehat{\delta }}_{t}^{*}=\psi {Y}_{t}^{*}+(1-\psi ){\widehat{\delta }}_{t-1}^{*}$$

The $$\psi \in \left[ {0,1} \right]$$ indicates the SC value which is used to get an estimate of the process CV shift $$\hat{\delta }_{t}^{*}$$ size at $$t$$ time sample value. If $${\widehat{\delta }}_{t}^{*}=0$$ the respective shift size $${\varvec{\delta}}$$ estimation is unbiased because the process is in phase I which is an in-control process state. But in real practice, the estimated shift $${\widehat{\delta }}_{t}^{*}$$ gives biased results as $${\widehat{\delta }}_{t}^{*}\ne 0.$$ For this, Haq and Khoo^27^ gave an unbiased procedure to estimate shift size through $${\widehat{\delta }}_{t}^{**}$$ estimator. Hence, Haq and Khoo^27^ suggested a way to precisely estimate the process CV shift magnitude $${\varvec{\delta}}$$, provided as follows:

2 $${\widehat{\delta }}_{t}^{**}=\frac{{\widehat{\delta }}_{t}^{*}}{1-{(1-\psi )}^{t}}$$

The estimated shift $${\widehat{\delta }}_{t}^{**}$$ expectation is $${E(\widehat{\delta }}_{t}^{**})={\varvec{\delta}}$$ . For no process shift, the process is assumed with the zero expectations as $${E(\widehat{\delta }}_{t}^{**})={\varvec{\delta}}=0$$ at time $$t\le {t}_{0}$$, it strengthens the argument that the process has no shift but as the process encountered some shift, the expectation becomes $${E(\widehat{\delta }}_{t}^{**})={\varvec{\delta}}\ne 0$$ at time $$t>{t}_{0}$$. Further, the CV shift is assumed to be estimated with the conditions: $${\widehat{\delta }}_{t}^{**}>0$$ or $${\widehat{\delta }}_{t}^{**}<0$$ for the $${\widehat{\delta }}_{t}^{*}>0$$ or $${\widehat{\delta }}_{t}^{*}<0$$, for the negative or the positive value respectively. Here, it is important that the $${\widetilde{\delta }}_{t}$$ is suggested to be estimated positively for $${\widehat{\delta }}_{t}^{**}$$ as $${\widetilde{\delta }}_{t}=\left|{\widehat{\delta }}_{t}^{**}\right|$$. The respective $$\left|{\widehat{\delta }}_{t}^{**}\right|$$ is suitable for all magnitudes for the occurring process CV shift $${\varvec{\delta}}$$ and noted as $${\varvec{\delta}}{\gamma }_{t=0}.$$

The presented AAEWMA CV chart statistic is ${\mathcal{A}}_{t}$(mentioned in the equation ) which is developed by Sarwar and Noor-ul-Amin^29^. It opts opting the sequence of computed CV values as $\left\{{Y}_{t}^{*}\right\}$ by using the data from a random sample of size $n$. The suggested ${\mathcal{A}}_{t}$ is an optimized way to efficiently and precisely identify any parametric increase or decrease in the process CV for any size of the increase or decrease ${\varvec{\delta}}$ shifts. The recursively calculated EWMA statistic ${\mathcal{A}}_{t}$ is used as a plotting statistic. The recursively sequenced $\left\{{\mathcal{A}}_{t}\right\}$ values deal in two ways: with an increase ${\varvec{\delta}}$ in the process CV is taken as a negative sequence of $\left\{{\mathcal{A}}_{t}^{-}\right\}$ or decrease ${\varvec{\delta}}$ as the positive sequence of $\left\{{\mathcal{A}}_{t}^{+}\right\}$ values, and then $\left\{{\mathcal{A}}_{t}\right\}$ as plotting statistics point toward the lower control limit toward the upper control limit or towards both limits of the control chart for both types of shifts. In this theory, three types of control charts are constructed: to investigate both sides as increase or decrease ${\varvec{\delta}}$ shifts and taken as a two-sided chart with $\left\{{\mathcal{A}}_{t}^{-}\right\}$ and $\left\{{\mathcal{A}}_{t}^{+}\right\}$ plotting statistics (determined results are shown in Table 1), and two one-sided charts (mentioned in Table 2 and Table 3); one to monitor the positive value of the statistic $\left\{{\mathcal{A}}_{t}^{+}\right\}$ and negative values of $\left\{{\mathcal{A}}_{t}^{-}\right\}$ statistic to investigate both decrease and increase ${\varvec{\delta}}$ shifts separately.

The proposed design methodology shows that the suggested AAEWMA CV chart operates as a two-sided monitoring control chart to investigate both increase and decrease shifts simultaneously through a single control chart statistic and two one-sided monitoring control charts to analyze the process increase and decrease in parametric CV ${\varvec{\delta}}$ shift, the plotting statistic is mentioned as follows:

3 $${\mathcal{A}}_{t}=\mathfrak{F}\left({\widetilde{\gimel }}_{t}^{*}\right){Y}_{t}^{*}+(1-\mathfrak{F}\left({\widetilde{\gimel }}_{t}^{*}\right)){\mathcal{A}}_{t-1}$$

In the above equation, $\mathfrak{F}\left({\widetilde{\gimel }}_{t}^{*}\right)$ is the respective function originally designed by Sarwar and Noor-ul-Amin^29^ and it gives better adapted SC value in the range ${\mathfrak{F}}\left( {\tilde{\gimel }_{t}^{*} } \right) \in$ (0, 1]is specified.

At the start of the process, the SC value is supposed to be ${\mathcal{A}}_{0} = 0$ for the computation of the first value of the plotting statistic ${\mathcal{A}}_{t}$. The ${\mathcal{A}}_{t}$ computations are made by using the SC values which are roughly optimized and computed through $\mathfrak{F}\left({\widetilde{\gimel }}_{t}^{*}\right)$, proposed by Sarwar and Noor-ul-Amin^29^ which makes the plotting statistic ${\mathcal{A}}_{t}$ of the proposed concept a capable statistic to effectively detect the change in the process of CV ${\varvec{\delta}}$ shifts. The roughly optimized approach is a subjectively optimized approach to get the SC value using the function $\mathfrak{F}\left({\widetilde{\gimel }}_{t}^{*}\right)$ computed or determined the estimated CV shift ${\varvec{\delta}}$ as ${\widetilde{\delta }}_{t}$ shift size, given as:

4 $$\mathfrak{F}\left({\widetilde{\gimel }}_{t}^{*}\right)=\left\{\begin{array}{c}\frac{1}{a\left[1+{\left({\widehat{\delta }}_{t}\right)}^{-c}\right]} if 0.00<{\widetilde{\delta }}_{t}\le 2.7\\ 1 if {\widetilde{\delta }}_{t}\ge 2.7\end{array}\right.$$

The entity $$\mathfrak{F}\left({\widetilde{\gimel }}_{t}^{*}\right)$$ introduced by Sarwar and Noor-ul-Amin^29^ to make computed statistic efficient, operates with a constant $$a$$ as $$a=7$$ and constant ***c*** is suggested to have 2 and 1 for the estimated shift sizes as $${\widehat{\delta }}_{t}\le 1$$ and $$1<{\widehat{\delta }}_{t}\le 2.7$$ respectively. As the value $${\widetilde{\delta }}_{t}$$ the computed shift becomes 2.7 as $${\widetilde{\delta }}_{t}\ge 2.7$$ then the AAEWMA CV chart becomes the Shewhart EWMA CV monitoring chart. Importantly, $${\mathcal{A}}_{t}$$ the statistic is a continuous type of random variable and $${\mathcal{A}}_{t}$$ is determined using the optimal SC which is computed by $$\mathfrak{F}\left({\widetilde{\gimel }}_{t}^{*}\right)$$ (computed from Eq. (4)) and then use $$\mathfrak{F}\left({\widetilde{\gimel }}_{t}^{*}\right)$$ in the computation of EWMA statistic as $${\mathcal{A}}_{t}$$ (computed from Eq. (3)), to get a rapid detection of process CV shift as $$\left|{\widehat{\delta }}_{t}^{**}\right|$$ within the specified given ranges as $$0.00<{\widetilde{\delta }}_{t}\le 2.7$$ so $${\widetilde{\delta }}_{t}$$ becomes as $${\widetilde{\delta }}_{t}=\left|{\widehat{\delta }}_{t}^{**}\right|$$. The SC computation through $$\mathfrak{F}\left({\widetilde{\gimel }}_{t}^{*}\right)$$ function and then use it for the proposed $${\mathcal{A}}_{t}$$ statistic calculation is the novelty of the presented paper and given by Sarwar and Noor-ul-Amin^29^ The efficiency of the suggested $$\mathfrak{F}\left({\widetilde{\gimel }}_{t}^{*}\right)$$ triggered the authors to present an improved and efficient design of the CV AAEWMA control chart by using the novel approach and presenting it in the underlying manuscript.

**Decision rule:** the decision rule to get a two-sided proposed AAEWMA CV control chart to indicate an out-of-control detection: $\left|{\mathcal{A}}_{t}\right|>\mathfrak{h}$ (${\mathcal{A}}_{t}^{+}>\mathfrak{h}$ and ${\mathcal{A}}_{t}^{+}<-\mathfrak{h})$**,** otherwise vice versa. For the respective one-sided proposed decreased shift detection, the AAEWMA CV chart goes out of control when ${\mathcal{A}}_{t}>\mathfrak{h}$ with the decrease or the increase process CV ${\varvec{\delta}}$ shift when ${\mathcal{A}}_{t}<-\mathfrak{h}$ or vice versa.

The constant $\mathfrak{h}$ is a fixed threshold value which is a positive invariant regarded as $\mathfrak{h}>0$ and identifies the limit of the specified control chart with a specified fixed in-control ARL threshold value to control the process, determined for the proposed AAEWMA CV statistic $\left|{\mathcal{A}}_{t}\right|$.

## Performance evaluation

This section uses the ARL and SDRL RL values to evaluate the proposed AAEWMA CV control chart's performance. The literature on SPC uses a variety of simulation techniques, including integral equation, Markov chain, and MC simulation techniques. Amongst these, MC simulation is the most widely used method in SPC studies. Tables 1, 2, 3 showcase the RL characteristics derived from the MC simulations in this study and unveil distinct predicted shifts across 100,000 replications within each iteration of 100,000 runs. Table 1 illustrates a two-sided control chart while Tables 2 and 3 depict the results for one-sided control charts which emphasize increased and decreased shift detections respectively. When the direction of the process shift is unclear, the corresponding two-sided control chart is the recommended method for the upward (increased magnitude of shift) and downward movement (decreased magnitude of shift) of the plotting statistic. The respective two-sided chart allows liberty to move ${\mathcal{A}}_{t}$ pointing towards both dimensions (upward or downward). The computed findings show that the ARL values of the two-sided AAEWMA CV chart are greater than those of the other two one-sided charts. The reason behind this is that whereas one-sided charts are intended for known cases, two-sided charts are advised for plotting unknown statistics. For this reason, it is not advised to use either of the chart types for the interim comparison. However, the preferable assumption is to get prior phase I data about the process shift magnitude, whether it might be a decreased or increased shift, to have a sensitive detection of the process shift. If not, a two-sided chart is a superior option when compared to its counterparts.

The parametric values $n = 3, 5, 8,10$, with $\gamma_{0} = 0.10, 0.15, 0.20,$ are considered to determine the results of the proposed chart. The samples are drawn from the normal distribution and the shifts ${\varvec{\delta}} = \left( {0.25, \ldots ,0.90,0.95,1.00,1.05,1.10, \ldots ,3.00} \right)$ are taken whereas the in-control threshold is fixed as the $ARL_{0} \cong 370$.

As stated in Eq. (1), the SC for the estimation of ${\varvec{\delta}}$ shifts is based on the computation of the process CV ${\varvec{\delta}}$ size using the EWMA statistic, so the assumed value of SC is ψ = 0.1. It is also possible to take into account and use the other SC values to identify minor to moderate shifts.

In this case, the threshold value for the two-sided AAEWMA CV chart determined is  = 0.3379, whereas  = 0.1946 at $\psi =0.1$ is the value for the one-sided rise and one-sided decrease shift ${\varvec{\delta}}$ detection.

The following are the findings of the computed results:

(i) For the constant values of $\psi$, ${\gamma }_{0}$ and ${\varvec{\delta}}$, the ${ARL}_{1}$ and the ${SDRL}_{1}$ decrease as the value of sample size $n$ increases, (detail is mentioned in Table 1), For instance, at decreased shift ${\varvec{\delta}} = 0.90$, $\gamma_{0} = 0.10$, $\psi = 0.1$ for $n = 3, 5, 8, 10$ the $ARL_{1} \cong \left( {95.17, 55.74,34.79,28.05} \right)$ and the $SDRL_{1} \cong \left( {76.32,43.15,27.34,22.19} \right)$. Moreover, at increased shift ${\varvec{\delta}} = 1.10$ the increased shift, the respective $ARL_{1} \cong \left( {78.24,48.19,32.26,26.36} \right)$ and the $SDRL_{1} \cong \left( {72.95,43.56,28.44,22.96} \right)$. There is a decreasing trend in $ARL_{1}$ and $SDRL_{1}$ values as sample size $n$ increases.
(ii) The behavior of respective RL shows a constant trend in $ARL_{1}$ and $SDRL_{1}$ for the same $\psi$, $n,$ and ${\varvec{\delta}}$, for any value of $\gamma_{0}$. From Table 1 study it exhibits that with ${\varvec{\delta}} = 0.90$ and $n = 5$ at $\gamma_{0} = 0.10, 0.15, 0.20$, the $ARL_{1} \cong \left( {55.74,55.53,55.65} \right)$ and the $SDRL_{1} \cong \left( {43.15,43.45,44.58} \right)$. The same can be observed at ${\varvec{\delta}} = 1.10$ the $ARL_{1} \cong \left( {48.19, 48.63, 50.11} \right)$ and the $SDRL_{1} \cong \left( {43.56,43.56, 44.77} \right)$. The computed result remains the same and any value of $\gamma_{0}$ (small or large) will not affect the trend of the computed results of $ARL_{1}$ and $SDRL_{1}$ values.
(iii) For the constant parametric values of $\psi$, $\gamma_{0}$ and $n$, the respective $ARL_{1}$ and $SDRL_{1}$ shows a decreasing trend as shift ${\varvec{\delta}}$ size in process CV increases or decreases. Table 1 shows that a two-sided control chart with $\gamma_{0} = 0.10$ and $n = 5$, at ${\varvec{\delta}} = 0.95, 0.90, 0.55, 0.50$ the $ARL_{1} \cong \left( {133.80,55.74,3.78,3.13} \right) {\text{and}} SDRL_{1} \cong \left( {118.00,43.15,1.77,1.30} \right)$. For ${\varvec{\delta}} = 1.05, 1.10, 1.20, 1.30$ the $ARL_{1} \cong \left( {116.34,48.19,17.15,9.17} \right) {\text{and}} SDRL_{1} \cong \left( {111.94,43.56,15.32,7.89} \right)$.
(iv) The one-sided respective study of the proposed chart results revealed that at the fixed parametric $\psi$, $\gamma_{0}$ and ${\varvec{\delta}}$, as the $n$ value increases, the RL values from Table 2 show an instantly decrease trend as $ARL_{1}$ and $SDRL_{1}$. For example, at ${\varvec{\delta}} = 1.10$, $\gamma_{0} = 0.10$ and $\psi = 0.1$ at the various values of sample size $n = 3, 5, 8, 10$ the $ARL_{1} \cong \left( {47.27,29.08,18.85,15.84} \right)$ and the $SDRL_{1} \cong \left( {54.18,32.70,20.75.17.43} \right)$. Just like that at ${\varvec{\delta}} = 0.90$ to study the decreased shift in process CV from Table 3$ARL_{1} \cong \left( {51.08, 30.29,19.13,15.62} \right)$ and the $SDRL_{1} \cong \left( {52.59,30.60,19.04,15.54} \right)$. Hence, the trend is in strong favor of the reasonable selection of the sample size in getting precise, efficient, and sensitive results.
(v) For the parametric settings $$\psi$$, $$\gamma_{0}$$ and $$ n$$, as shift $${\varvec{\delta}}$$ increases in process CV, the RL values of $$ARL_{1}$$ and $$SDRL_{1}$$ decrease rapidly which are mentioned in Tables 2 and Table 3, So, results at $${\varvec{\delta}} = 1.05,1.10, {\text{and}} 0.95, 0.90$$ for $$\gamma_{0} = 0.10$$, $$\psi = 0.1$$ at sample size $$n = 5,$$ revealed the RL values: $$ARL_{1} \cong \left( {67.18,29.08, {\text{and }}71.56,30.29} \right)$$ and $$SDRL_{1} \cong \left( {77.00,32.70, {\text{and }}77.94,30.60} \right)$$. Likewise, at the sample size $$n = 10$$ the RL values are $$ARL_{1} \cong \left( {41.28,15.84, {\text{and}} 42.50,15.62} \right)$$ and the $$SDRL_{1} \cong \left( {45.94, 17.43, {\text{and}} 45.07,15.54} \right)$$.

**Figure 1:**
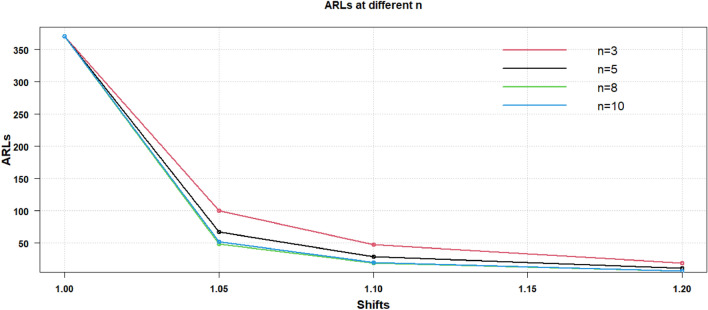
Proposed AAEWMA CV control chart for ${\gamma }_{0}=0.10$.

**Figure 2:**
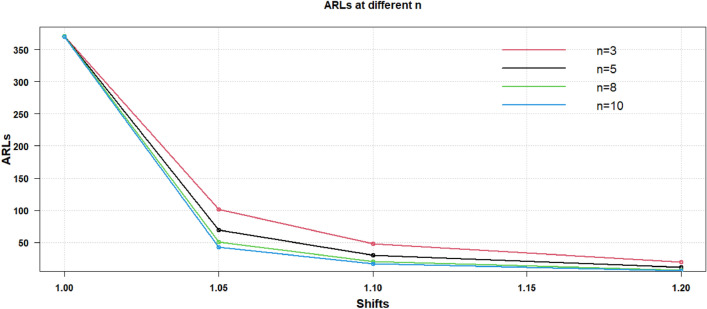
Proposed AAEWMA CV control chart for ${\gamma }_{0}=0.20$.

Thus, above mentioned results, the discussion revealed that for any size of increased or decreased shift in process CV sample size is the criteria. It can be concluded that the parameter for enhancing the efficacy of the suggested CV chart is $n$. The same is shown through the graphical display in Figs. 1, 2 of the results provided in Table 2. In Fig. 1, the results for various sample sizes like $n = 3,5,8,10$ are provided for $\gamma_{0} = 0.10$ and in Fig. 2, the results are displayed for the $\gamma_{0} = 0.20$ for the change in increased process CV shifts.

## Comparitive analysis

A comparison is made with the current AEWMA CV control chart provided by Haq and Khoo^27^ is done in this part of the manuscript. It is claimed by Haq and Khoo^27^ that the AEWMA CV^27^ outperforms its competitors in detecting moderate to large process shifts and is considered the most effective AEWMA chart for monitoring any type of shift at the earliest stage.

This section provides an overview by using the following parameters: $n = 3, 5, 8, 10$; $\gamma_{0} = 0.10$; $\psi = 0.1$ for the control threshold as $ARL_{0} \cong 370$. Tables 4, 5, 6 present the comparative analysis of the one-sided and two-sided increase and decrease CV shift values, taking into account typical parametric settings to elaborate the comprehensively determined results.

**Table 4:** Comparative Analysis of the proposed AAEWMA CV chart two-sided RL profile for $ARL_{0} \cong 370$ with Existing AEWMA CV Chart.

${\varvec{\delta}}$	Comparitive study between existing AEWMA CV & Proposed AAEWMA CV two sided with $\psi$ $= 0.1,$ ${\mathbf{\mathfrak{h}}} =$ 0.7130 & ${\mathbf{\mathfrak{h}}} =$ 0.3379																
		**Existing** AEWMA CV $\gamma_{0} = 0.10$				**Proposed** AAEWMA CV $\gamma_{0} = 0.10$				**Existing** AEWMA CV $\gamma_{0} = 0.20$				**Proposed** AAEWMA CV $\gamma_{0} = 0.20$			
		$n = 3$	$n = 5$	$n = 8$	$n = 10$	$n = 3$	$n = 5$	$n = 8$	$n = 10$	$n = 3$	$n = 5$	$n = 8$	$n = 10$	$n = 3$	$n = 5$	$n = 8$	$n = 10$
0.25	${\varvec{ARL}}_{1}$	1.55	1.01	1.00	1.00	2.47	1.60	1.05	1.00	1.55	1.01	1.00	1.00	2.48	1.61	1.05	1.00
	$SDRL_{1}$	0.86	0.11	0.00	0.00	0.75	0.50	0.22	0.05	0.86	0.11	0.00	0.00	0.75	0.50	0.23	0.06
0.50	${\varvec{ARL}}_{1}$	4.89	2.16	1.32	1.13	5.92	3.13	2.09	1.80	4.86	2.21	1.33	1.14	5.98	3.17	2.12	1.82
	$SDRL_{1}$	3.70	1.43	0.66	0.40	3.15	1.30	0.70	0.59	3.71	1.49	0.69	0.43	3.21	1.33	0.72	0.60
0.55	${\varvec{ARL}}_{1}$	6.41	2.77	1.60	1.31	7.38	3.78	2.45	2.07	6.39	2.81	1.61	1.34	7.49	3.85	2.50	2.10
	$SDRL_{1}$	5.34	1.94	0.98	0.66	4.32	1.77	0.93	0.73	5.48	1.97	0.98	0.70	4.38	1.83	0.95	0.75
0.60	${\varvec{ARL}}_{1}$	8.85	3.61	2.02	1.63	9.52	4.72	2.95	2.48	8.97	3.68	2.07	1.66	9.60	4.77	3.00	2.51
	$SDRL_{1}$	8.46	2.72	1.38	1.01	6.02	2.48	1.28	0.98	8.57	2.77	1.41	1.04	6.12	2.51	1.31	1.01
0.65	${\varvec{ARL}}_{1}$	13.00	4.96	2.68	2.10	12.64	6.10	3.69	3.03	13.26	4.96	2.75	2.15	12.66	6.18	3.76	3.11
	$SDRL_{1}$	13.37	4.13	1.95	1.46	8.51	3.56	1.81	1.36	13.66	4.16	2.02	1.52	8.61	3.61	1.87	1.42
0.70	${\varvec{ARL}}_{1}$	20.04	7.16	3.68	2.89	17.06	8.19	4.86	3.91	20.57	7.35	3.80	2.96	17.22	8.43	4.94	4.02
	$SDRL_{1}$	21.71	6.58	2.90	2.18	12.14	5.32	2.71	2.02	22.09	6.99	3.07	2.25	12.19	5.48	2.80	2.08
0.75	${\varvec{ARL}}_{1}$	31.86	11.38	5.58	4.13	24.22	11.58	6.75	5.37	31.66	11.69	5.63	4.33	24.60	11.90	6.92	5.53
	$SDRL_{1}$	34.39	11.87	5.01	3.43	17.86	8.12	4.25	3.21	34.02	12.23	5.10	3.63	17.98	8.32	4.45	3.34
0.80	${\varvec{ARL}}_{1}$	49.26	20.09	9.40	6.85	35.63	17.76	10.18	8.05	50.34	20.50	9.72	6.97	35.89	18.27	10.55	8.30
	$SDRL_{1}$	52.92	21.67	9.60	6.67	26.62	13.14	7.17	5.44	52.84	22.35	10.00	6.80	26.90	13.55	7.49	5.62
0.85	${\varvec{ARL}}_{1}$	77.47	36.97	18.82	13.85	55.29	29.69	17.37	13.82	79.50	38.16	20.03	14.33	56.02	30.35	18.07	14.20
	$SDRL_{1}$	81.93	39.82	20.61	15.17	42.02	22.64	13.21	10.32	83.35	41.24	22.14	15.52	42.68	23.19	13.76	10.66
0.90	${\varvec{ARL}}_{1}$	136.19	72.99	43.17	32.93	95.17	55.74	34.79	28.05	137.64	73.81	43.70	34.24	95.19	56.65	35.82	29.07
	$SDRL_{1}$	150.13	77.82	46.64	35.61	76.32	43.15	27.34	22.19	149.76	80.05	47.22	37.26	76.84	44.58	28.24	22.78
0.95	${\varvec{ARL}}_{1}$	267.75	177.50	120.10	98.45	202.74	133.80	91.53	77.06	268.31	183.41	124.32	103.86	204.16	136.25	94.96	79.19
	$SDRL_{1}$	315.33	208.21	134.48	110.13	190.17	118.00	76.92	64.01	313.10	212.52	141.36	115.39	193.93	120.00	79.50	65.37
1.00	${\varvec{ARL}}_{0}$	366.61	370.02	371.31	365.74	372.91	374.18	374.24	374.59	367.42	368.51	372.20	376.18	372.69	368.31	372.65	373.59
	$SDRL_{0}$	463.39	474.19	460.12	451.04	407.30	407.74	408.20	410.12	460.32	461.73	463.88	466.17	408.87	405.73	410.58	409.42
1.05	${\varvec{ARL}}_{1}$	181.01	129.89	92.18	77.28	168.25	116.34	82.59	70.49	184.30	131.03	95.29	80.78	170.48	117.88	85.25	73.45
	$SDRL_{1}$	222.17	159.83	108.53	90.36	171.35	111.94	74.85	63.44	229.47	159.59	113.97	96.59	172.22	112.63	77.45	65.41
1.10	${\varvec{ARL}}_{1}$	82.42	48.82	31.59	25.18	78.24	48.19	32.26	26.36	83.19	50.42	32.15	26.37	79.88	50.11	32.95	27.21
	$SDRL_{1}$	98.85	58.22	35.84	29.05	72.95	43.56	28.44	22.96	100.94	59.95	37.05	30.69	75.02	44.77	28.99	23.69
1.20	${\varvec{ARL}}_{1}$	28.23	15.24	8.87	6.93	30.76	17.15	10.72	8.58	28.72	15.58	9.51	7.31	31.52	17.91	11.23	9.05
	$SDRL_{1}$	33.56	18.03	10.07	7.63	28.18	15.32	9.14	7.09	34.23	18.40	10.90	8.07	28.95	16.06	9.56	7.49
1.30	${\varvec{ARL}}_{1}$	14.07	7.34	4.30	3.52	16.63	9.17	5.77	4.71	14.52	7.55	4.61	3.73	17.17	9.53	6.01	4.95
	$SDRL_{1}$	16.94	8.42	4.35	3.34	15.41	7.89	4.51	3.47	17.42	8.58	4.77	3.67	15.92	8.34	4.73	3.68
1.40	${\varvec{ARL}}_{1}$	8.67	4.49	2.83	2.33	10.86	5.97	3.84	3.24	8.81	4.69	2.97	2.47	11.10	6.26	4.06	3.37
	$SDRL_{1}$	10.30	4.69	2.55	1.95	9.90	4.81	2.72	2.14	10.65	4.96	2.76	2.11	10.06	5.14	2.93	2.27
1.50	${\varvec{ARL}}_{1}$	5.88	3.30	2.13	1.83	7.83	4.40	2.91	2.48	6.18	3.38	2.25	1.90	8.05	4.61	3.06	2.58
	$SDRL_{1}$	6.73	3.18	1.73	1.38	6.95	3.36	1.91	1.49	7.15	3.25	1.90	1.47	7.13	3.59	2.02	1.58
1.60	${\varvec{ARL}}_{1}$	4.58	2.58	1.78	1.52	6.06	3.48	2.37	2.05	4.73	2.68	1.84	1.59	6.24	3.64	2.50	2.14
	$SDRL_{1}$	4.97	2.36	1.30	0.98	5.20	2.52	1.43	1.15	5.24	2.43	1.38	1.08	5.39	2.68	1.54	1.23
1.70	${\varvec{ARL}}_{1}$	3.69	2.17	1.54	1.34	4.94	2.91	2.04	1.77	3.76	2.25	1.59	1.40	5.12	3.06	2.14	1.85
	$SDRL_{1}$	3.83	1.81	1.02	0.75	4.11	1.98	1.15	0.92	3.99	1.91	1.08	0.83	4.28	2.10	1.24	0.99
1.80	${\varvec{ARL}}_{1}$	3.10	1.88	1.38	1.23	4.18	2.52	1.81	1.57	3.21	1.98	1.43	1.27	4.33	2.64	1.88	1.64
	$SDRL_{1}$	3.00	1.47	0.80	0.59	3.34	1.65	0.98	0.78	3.23	1.57	0.86	0.65	3.53	1.78	1.02	0.81
1.90	${\varvec{ARL}}_{1}$	2.72	1.69	1.27	1.16	3.65	2.25	1.62	1.44	2.79	1.77	1.32	1.20	3.78	2.34	1.70	1.49
	$SDRL_{1}$	2.54	1.21	0.65	0.46	2.86	1.40	0.82	0.66	2.70	1.33	0.71	0.54	2.96	1.49	0.89	0.71
2.00	${\varvec{ARL}}_{1}$	2.42	1.55	1.20	1.11	3.22	2.03	1.50	1.33	2.52	1.61	1.24	1.14	3.34	2.13	1.57	1.39
	$SDRL_{1}$	2.17	1.04	0.54	0.38	2.44	1.22	0.72	0.57	2.31	1.12	0.61	0.44	2.56	1.30	0.78	0.62
2.25	${\varvec{ARL}}_{1}$	1.70	1.22	1.06	1.03	2.20	1.48	1.18	1.10	1.77	1.27	1.08	1.04	2.29	1.56	1.22	1.13
	$SDRL_{1}$	1.24	0.57	0.26	0.16	1.48	0.73	0.42	0.31	1.35	0.65	0.30	0.20	1.56	0.80	0.47	0.36
3.00	${\varvec{ARL}}_{1}$	1.43	1.11	1.02	1.01	1.77	1.27	1.07	1.03	1.47	1.14	1.03	1.01	1.85	1.33	1.11	1.05
	$SDRL_{1}$	0.89	0.37	0.15	0.08	1.06	0.53	0.27	0.18	0.94	0.43	0.19	0.11	1.14	0.60	0.33	0.23

**Table 5:** Comparative Analysis of the proposed AAEWMA CV chart one-sided increase CV shift RL profile for $ARL_{0} \cong 370$.

	Comtive study between existing AEWMA CV & Proposed AAEWMA CV one-sided increase CV shift with $\psi$ $= 0.1,$ ${\mathbf{\mathfrak{h}}}$ = 0.6125 & ${\mathbf{\mathfrak{h}}}$ = 0.1946																
		**Existing** AEWMA CV $\gamma_{0} = 0.10$				**Proposed** AAEWMA CV $\gamma_{0} = 0.10$				**Existing** AEWMA CV $\gamma_{0} = 0.20$				**Proposed** AAEWMA CV $\gamma_{0} = 0.20$			
		$n = 3$	$n = 5$	$n = 8$	$n = 10$	$n = 3$	$n = 5$	$n = 8$	$n = 10$	$n = 3$	$n = 5$	$n = 8$	$n = 10$	$n = 3$	$n = 5$	$n = 8$	$n = 10$
1.00	${\varvec{ARL}}_{0}$	370.57	370.41	370.57	370.34	370.74	370.34	370.93	370.47	370.28	370.57	370.42	370.81	370.92	370.71	370.82	370.12
	$SDRL_{0}$	423.46	417.90	424.97	412.50	470.55	467.07	470.32	470.15	413.34	423.08	413.30	418.89	467.89	461.82	468.22	464.40
1.05	${\varvec{ARL}}_{1}$	120.00	84.12	61.64	52.34	100.13	67.18	48.71	41.28	121.53	86.97	63.54	54.04	101.28	69.14	50.44	42.74
	$SDRL_{1}$	132.29	91.07	65.79	55.38	117.48	77.00	54.53	45.94	132.70	93.87	67.21	55.95	119.95	79.24	56.57	47.71
1.10	${\varvec{ARL}}_{1}$	57.86	36.08	23.95	19.69	47.27	29.08	18.85	15.84	59.11	37.61	24.97	20.74	48.03	29.88	19.84	16.34
	$SDRL_{1}$	62.27	38.58	24.67	20.42	54.18	32.70	20.75	17.43	63.10	38.99	26.17	21.48	55.10	33.61	22.07	17.86
1.20	${\varvec{ARL}}_{1}$	23.05	12.73	7.97	6.19	19.24	10.70	6.72	5.59	23.38	13.43	8.22	6.65	19.43	11.13	7.10	5.85
	$SDRL_{1}$	24.77	13.84	8.58	6.46	22.08	11.97	6.91	5.53	25.23	14.50	8.80	7.00	22.45	12.35	7.40	5.83
1.30	${\varvec{ARL}}_{1}$	12.43	6.67	4.07	3.30	10.73	5.98	3.82	3.16	12.82	7.02	4.28	3.48	10.97	6.22	4.01	3.32
	$SDRL_{1}$	13.78	7.25	4.07	3.14	12.18	6.22	3.49	2.70	14.08	7.62	4.30	3.30	12.33	6.53	3.75	2.89
1.40	${\varvec{ARL}}_{1}$	7.95	4.28	2.68	2.27	7.12	4.01	2.67	2.27	8.20	4.48	2.85	2.33	7.40	4.16	2.79	2.36
	$SDRL_{1}$	8.83	4.36	2.37	1.87	7.83	3.86	2.14	1.69	9.05	4.71	2.55	1.94	8.06	4.07	2.30	1.78
1.50	${\varvec{ARL}}_{1}$	5.68	3.11	2.07	1.77	5.28	3.06	2.08	1.79	5.87	3.26	2.15	1.85	5.37	3.18	2.18	1.88
	$SDRL_{1}$	6.23	2.99	1.63	1.26	5.57	2.73	1.51	1.16	6.42	3.12	1.72	1.36	5.72	2.82	1.62	1.26
1.60	${\varvec{ARL}}_{1}$	4.40	2.48	1.72	1.49	4.18	2.49	1.76	1.54	4.54	2.59	1.79	1.56	4.31	2.58	1.82	1.61
	$SDRL_{1}$	4.64	2.20	1.20	0.92	4.26	2.03	1.14	0.87	4.83	2.30	1.28	1.02	4.36	2.13	1.21	0.96
1.70	${\varvec{ARL}}_{1}$	3.58	2.09	1.49	1.33	3.51	2.12	1.55	1.38	3.76	2.20	1.55	1.38	3.58	2.21	1.61	1.43
	$SDRL_{1}$	3.60	1.70	0.93	0.70	3.42	1.59	0.90	0.68	3.79	1.82	1.01	0.78	3.43	1.68	0.96	0.74
1.80	${\varvec{ARL}}_{1}$	3.06	1.83	1.36	1.22	2.99	1.89	1.40	1.27	3.19	1.93	1.41	1.26	3.08	1.95	1.45	1.31
	$SDRL_{1}$	2.98	1.34	0.77	0.56	2.75	1.31	0.73	0.55	3.15	1.50	0.81	0.62	2.84	1.40	0.78	0.61
1.90	${\varvec{ARL}}_{1}$	2.66	1.68	1.27	1.16	2.67	1.70	1.31	1.20	2.78	1.73	1.32	1.18	2.73	1.78	1.36	1.23
	$SDRL_{1}$	2.46	1.17	0.62	0.45	2.36	1.10	0.62	0.47	2.62	1.27	0.70	0.49	2.42	1.19	0.67	0.51
2.00	${\varvec{ARL}}_{1}$	2.37	1.54	1.20	1.11	2.38	1.58	1.24	1.14	2.47	1.61	1.24	1.14	2.48	1.64	1.28	1.17
	$SDRL_{1}$	2.12	1.00	0.53	0.37	1.98	0.95	0.54	0.39	2.24	1.09	0.59	0.41	2.11	1.03	0.58	0.44
2.25	${\varvec{ARL}}_{1}$	1.71	1.22	1.06	1.02	1.74	1.25	1.08	1.04	1.76	1.26	1.08	1.04	1.79	1.30	1.10	1.05
	$SDRL_{1}$	1.24	0.56	0.26	0.16	1.19	0.56	0.28	0.19	1.29	0.62	0.30	0.19	1.27	0.62	0.32	0.22
3.00	${\varvec{ARL}}_{1}$	1.43	1.11	1.02	1.01	1.46	1.14	1.03	1.01	1.47	1.14	1.03	1.01	1.51	1.17	1.04	1.02
	$SDRL_{1}$	0.88	0.38	0.15	0.08	0.85	0.39	0.17	0.10	0.94	0.43	0.18	0.11	0.91	0.44	0.21	0.13

**Table 6:** Comparative Analysis of the proposed AAEWMA CV chart one-sided decrease CV shift RL profile for $ARL_{0} \cong 370$.

${\varvec{\delta}}$	Comparitive study between existing AEWMA CV & Proposed AAEWMA CV one-sided decrease CV shift with $\psi$ $= 0.1,$ ${\mathbf{\mathfrak{h}}}$ = 0.6125 & ${\mathbf{\mathfrak{h}}}$ = 0.6125																
		Existing AEWMA CV $\gamma_{0} = 0.10$				Proposed AAEWMA CV $\gamma_{0} = 0.10$				Existing AEWMA CV $\gamma_{0} = 0.20$				Proposed AAEWMA CV $\gamma_{0} = 0.20$			
		$n = 3$	$n = 5$	$n = 8$	$n = 10$	$n = 3$	$n = 5$	$n = 8$	$n = 10$	$n = 3$	$n = 5$	$n = 8$	$n = 10$	$n = 3$	$n = 5$	$n = 8$	$n = 10$
1.00	${\varvec{ARL}}_{0}$	370.02	370.05	370.09	370.94	370.53	370.42	370.26	370.85	370.83	370.78	370.97	370.15	370.12	370.57	370.99	369.66
	$SDRL_{0}$	409.99	424.26	424.58	417.90	464.62	466.86	462.58	465.88	415.88	423.09	416.37	421.47	467.90	466.88	465.03	462.35
0.95	${\varvec{ARL}}_{1}$	148.28	100.26	69.95	58.58	108.31	71.56	50.48	42.50	145.26	101.89	72.61	60.39	108.30	72.67	51.78	43.79
	$SDRL_{1}$	159.50	104.42	71.37	58.50	119.88	77.94	53.43	45.07	157.65	105.94	73.19	60.95	121.10	79.10	55.06	46.70
0.90	${\varvec{ARL}}_{1}$	73.64	42.74	27.83	22.40	51.08	30.29	19.13	15.62	75.57	44.34	28.88	22.69	51.25	30.92	19.90	15.96
	$SDRL_{1}$	74.25	40.90	26.66	21.99	52.59	30.60	19.04	15.54	74.69	42.52	27.89	22.39	53.07	31.34	19.92	16.01
0.85	${\varvec{ARL}}_{1}$	43.54	23.74	13.69	10.47	29.64	16.34	9.94	7.95	44.74	24.66	14.22	10.92	29.60	16.68	10.21	8.22
	$SDRL_{1}$	41.11	22.91	13.56	10.59	29.37	15.75	9.23	7.16	42.19	23.53	14.34	10.89	29.31	16.29	9.56	7.50
0.80	${\varvec{ARL}}_{1}$	28.89	14.05	7.71	5.72	19.12	10.03	6.05	4.84	29.27	14.45	7.88	6.03	19.24	10.21	6.17	4.98
	$SDRL_{1}$	27.56	13.80	7.39	5.39	18.18	9.20	5.10	3.86	27.42	14.02	7.57	5.65	18.37	9.29	5.19	4.03
0.75	${\varvec{ARL}}_{1}$	20.16	9.06	4.74	3.68	13.17	6.78	4.11	3.33	20.62	9.22	4.95	3.80	13.36	6.92	4.22	3.43
	$SDRL_{1}$	19.52	8.70	4.17	2.98	12.07	5.63	3.03	2.30	19.53	9.03	4.33	3.10	12.32	5.89	3.17	2.40
0.70	${\varvec{ARL}}_{1}$	14.31	5.94	3.26	2.60	9.51	4.88	3.03	2.51	14.36	6.08	3.35	2.67	9.70	5.00	3.10	2.55
	$SDRL_{1}$	13.63	5.37	2.52	1.84	8.24	3.71	1.98	1.51	13.83	5.52	2.59	1.95	8.42	3.84	2.03	1.55
0.65	${\varvec{ARL}}_{1}$	10.00	4.26	2.43	1.97	7.11	3.68	2.35	1.99	10.07	4.30	2.48	2.00	7.18	3.77	2.41	2.04
	$SDRL_{1}$	9.47	3.43	1.67	1.26	5.68	2.51	1.35	1.05	9.37	3.51	1.72	1.29	5.81	2.58	1.40	1.08
0.60	${\varvec{ARL}}_{1}$	7.13	3.19	1.91	1.56	5.52	2.95	1.94	1.64	7.25	3.24	1.93	1.59	5.60	2.99	1.97	1.67
	$SDRL_{1}$	6.41	2.29	1.19	0.89	4.17	1.80	0.98	0.76	6.52	2.37	1.22	0.92	4.23	1.84	1.00	0.78
0.55	${\varvec{ARL}}_{1}$	5.34	2.50	1.53	1.29	4.43	2.40	1.62	1.39	5.43	2.52	1.56	1.31	4.44	2.42	1.64	1.41
	$SDRL_{1}$	4.35	1.66	0.85	0.60	3.05	1.30	0.73	0.57	4.43	1.69	0.87	0.63	3.04	1.32	0.74	0.59
0.50	${\varvec{ARL}}_{1}$	4.12	2.00	1.28	1.12	3.61	2.01	1.39	1.22	4.20	2.05	1.30	1.13	3.62	2.03	1.41	1.23
	$SDRL_{1}$	3.07	1.24	0.59	0.38	2.25	0.98	0.56	0.43	3.16	1.27	0.61	0.40	2.26	0.99	0.57	0.44
0.25	${\varvec{ARL}}_{1}$	1.47	1.01	1.00	1.00	1.61	1.05	1.00	1.00	1.48	1.01	1.00	1.00	1.62	1.05	1.00	1.00
	$SDRL_{1}$	0.74	0.09	0.00	0.00	0.61	0.22	0.01	0.00	0.75	0.11	0.00	0.00	0.61	0.23	0.01	0.00

For example, it is studied (see Table 4) that at $n = 3, 5, 8, 10,$ the two-sided results of both (proposed AAEWMA CV chart and the existing Haq and Khoo^27^) for the CV process shift as ${\varvec{\delta}} = 0.85$, gives $ARL_{1} \cong \left( {77.47,36.97,18.82,13.85} \right)$ for the existing Haq and Khoo^27^ whereas the proposed AAEWMA CV chart gives $ARL_{1} \cong \left( {55.29,29.69,17.37,13.82} \right)$.

In a similar vein, the existing AEWMA CV chart by Haq and Khoo^27^ yields $ARL_{1} \cong \left( {181.01,129.89,92.18,77.28} \right)$ as mentioned in Table 4 with an increase CV shift study ${\varvec{\delta}} = 1.05$, whereas the proposed AAEWMA CV chart provides $ARL_{1} \cong \left( {168.25,116.34,82.59,70.49} \right).$ Furthermore, the tabular study reveals that, in contrast to its counterparts, the current AEWMA CV chart is more effective for values of $n > 10$, but the suggested AAEWMA CV chart is effective for all values of *n* with any size, from small as 3 or 5 to large as 8 or 10. A similar trend of the proposed AAEWMA CV chart can be seen in Tables 5 and 6 for the one-sided increase and decrease CV shift tabular analysis.

**Figure 3:**
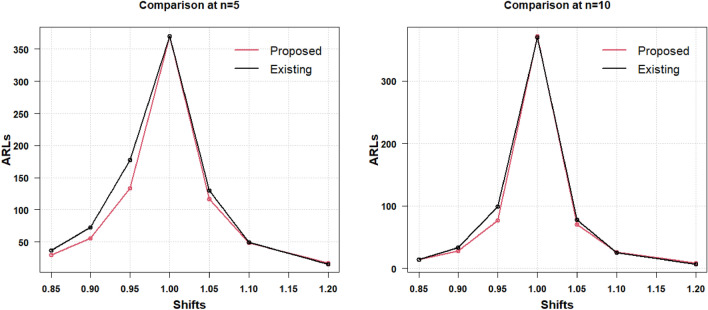
Increased and decreased shift comparison for ${\gamma }_{0}=0.10$ with $n=5$ and $n=10$.

**Figure 4:**
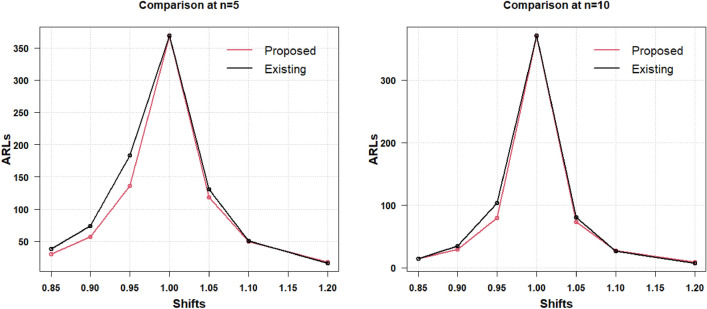
Increased and decreased shift comparison for ${\gamma }_{0}=0.20$ with $n=5$ and $n=10$.

**Figure 5:**
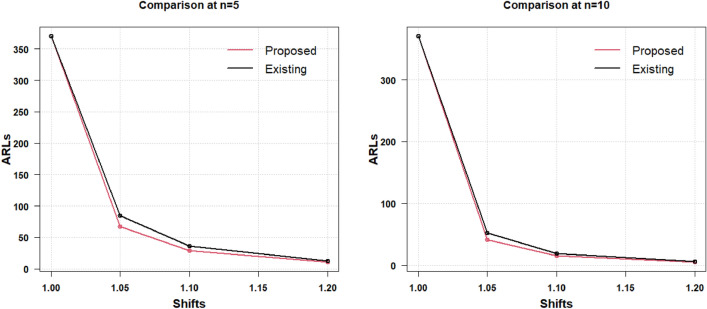
Increased shift comparative analysis for ${\gamma }_{0}=0.10$ with $n=5$ and $n=10$.

**Figure 6:**
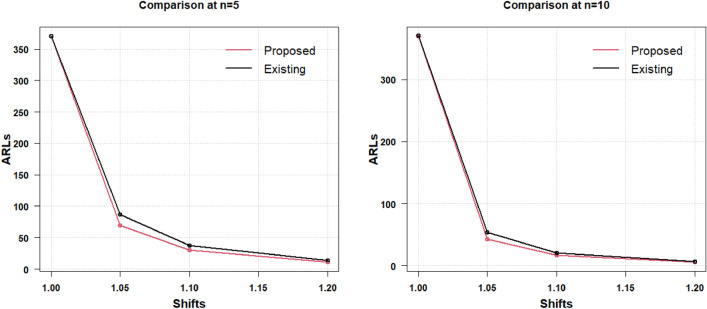
Increased shift comparative analysis for ${\gamma }_{0}=0.20$ with $n=5$ and $n=10$.

The presented AAEWMA CV control chart works effectively with smaller ARL values in the identification of small to moderate CV shifts, as was previously described. When the shift increases as 1.3 and decreases as 0.4, the two charts under discussion transform into Shewhart charts and display identical data. Therefore, it can be said that the suggested AAEWMA CV control chart is an effective CV chart. The efficiency level of the suggested CV chart remains unaffected by the change in $\gamma_{0}$, as was covered in the preceding section and is readily apparent from the tables. The results for the two-sided increased and decreased process CV shifts are graphically represented in Table 4, shown in Fig. 3 for the respective sample sizes $n = 5$ and $n = 10$ for $\gamma_{0} = 0.10$ and for $\gamma_{0} = 0.20$ in Fig. 4. The comparative picture for the increased CV shift results (from Table 5) has been discussed for the respective sample sizes $n = 5$ and $n = 10$ for $\gamma_{0} = 0.10$ in Fig. 5 and for $\gamma_{0} = 0.20$ in Fig. 6.

## Real life example

An industrial dataset from a sintering process which is reported by Castagliola et al.^16^ for the production of machine parts is used to demonstrate a real-world application. By compressing the metal powder after it has been heated to a specific temperature in a furnace. The sintering process is a powder metallurgy technique that creates the bonding of individual particles in the fabrication of mechanical parts. The sintering process is becoming more and more popular in machine tool manufacturing as a potentially cost-saving method compared to traditional manufacturing procedures. Since the sintering process depends on the furnace's temperature management, it can be accomplished by using the appropriate control chart to produce outputs that meet standards. The manufacturing businesses handling these operations promise a process drop time of ${T}_{pd}>30s$ (where $s$ is the unit of measurement for seconds) for bearings that range from 2 to 1.5 bars. The pore shrinkage capacity during the process, or ${Q}_{c}$, is the quality characteristic. Molten copper is applied to plug the pores and prolong, ${T}_{pd}$. for longer than 30 s.

The quantity of molten copper absorbed during the cooling process is denoted by ${Q}_{c}$. This quantity is directly correlated with the quantity of ${T}_{pd}$. The bigger the amount of copper consumed ${Q}_{c}$, the higher the anticipated ${T}_{pd}$. The process drop time standard deviation ${\sigma }_{pd}$ and the process drop time mean ${\mu }_{pd}$ can be stated as ${\sigma }_{pd}={\gamma }_{pd}*{\mu }_{pd}$; where ${\gamma }_{pd}$ is the process drop time CV. Castagliola et al.^16^ explained this constant proportional relation to track a dataset exhibiting consistent proportional behavior throughout time intervals between the process's mean and standard deviation. To effectively identify the rare process CV variations, it is advised to monitor such processes using CV control charts.

A proposed AAEWMA CV chart with one-sided monitoring is used to track the sintering dataset. Since the CV shift is greater, there is an increase in one direction. Thus, the example employs a one-sided increased CV shift chart technique. For phases I and II, two control charts suggested AAEWMA CV and the existing AEWMA CV, are designed with the in-control and out-of-control increased CV shift magnitude monitoring, respectively. The methodology will be implemented in real-life datasets like this:

Phase I: for each of $m=20$ sets of values, a random sample of size $n=5$ is drawn and the CV is computed as ${\widehat{\gamma }}_{i}=\frac{{S}_{i}}{{\overline{X} }_{i}}$; with $i = 1,2, \ldots , m$. Here, $S_{i}$ is the standard deviation and ${\overline{X} }_{i}$ is the mean of the process understudy. To determine the control limits, the phase I information is utilized to compute the in-control CV as ${\widehat{\gamma }}_{0}$ from $m$ values of phase I as ${\widehat{\gamma }}_{0}=\sqrt{\frac{\sum_{i=1}^{m}{\widehat{\gamma }}_{i}^{2}}{m}}=0.4170$.

Phase II: for in-control threshold ${ARL}_{0}\cong 370$ the value of $\mathfrak{h}=0.1946$ for the proposed AAEWMA CV and $\mathfrak{h}=0.6125$ for the existing counterpart AEWMA CV chart by Haq and Khoo^27^ for $\psi =0.1$. The CV process monitoring with shift size ${\varvec{\delta}}=1.25$; it means a $25 \%$ increase in Phase I CV ${\gamma }_{i}$ shift, computed as ${\gamma }_{1 }=1.25*{\widehat{\gamma }}_{0}=0.521$. Phase II involves the selection of further $m=20$ samples with $n=5$ size. The values of ${\widehat{\gamma }}_{i}$ CV is calculated to see the impact of the immediate shift size as ${\gamma }_{1.}$

**Figure 7:**
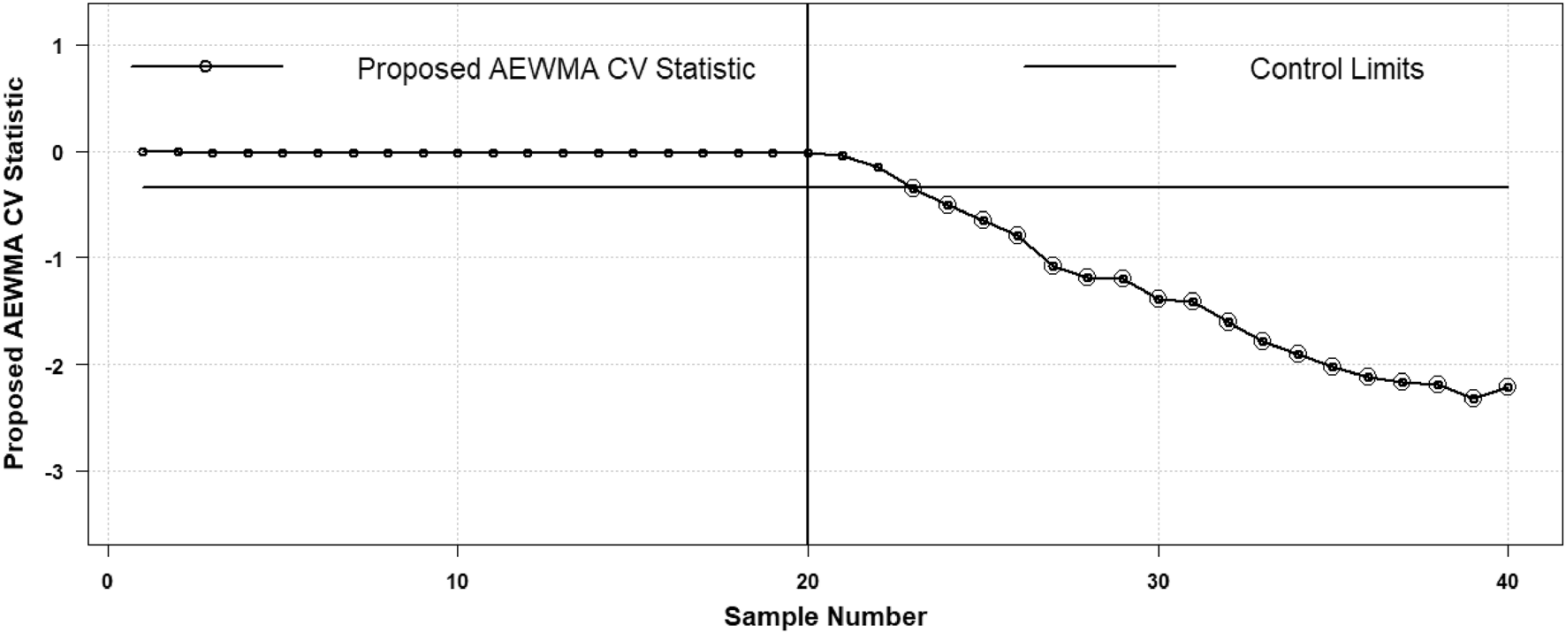
Proposed AAEWMA CV control chart (Real Data Based Example).

**Figure 8:**
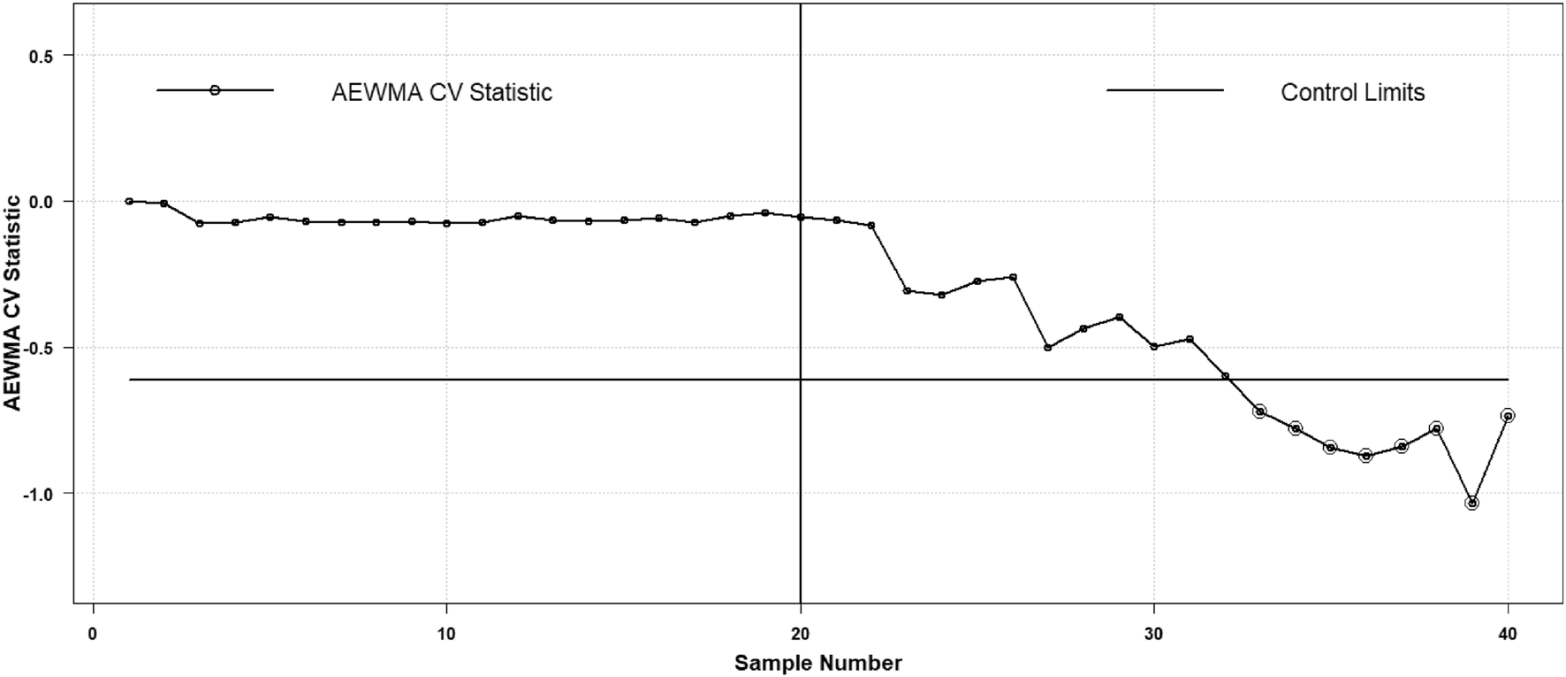
Existing AEWMA CV control chart (Real Data Based Example).

The plotting statistic ${H}_{t}$ of the existing AEWMA CV chart is calculated and the details can be seen in Haq and Khoo^27^. To execute the comparative analysis the proposed AAEWMA CV chart statistic ${\mathcal{A}}_{t}$ is also computed to show the performance of the proposed strategy. So, the two respective control charts are shown in Figs. 7 and 8. Both the plotting statistics ${\mathcal{A}}_{t}$ and ${H}_{t}$ computed values are also mentioned in Table 7 to elaborate on the values that show the out-of-control trend. From the charts, it is clear that the existing AEWMA CV chart provides detection at sample point 33^rd^, while the proposed AAEWMA CV chart provides detection at sample point 23^rd^. Therefore, it demonstrates that the suggested chart is more effective than its existing equivalent in identifying a 25% rise in CV.

**Table 7:** Graphing statistical values derived from density data employed in Figs. 1 and 2.

Sample #	Existing AEWMA CV	Proposed AAEWMA CV	Sample #	Existing AEWMA CV	Proposed AAEWMA CV
	Phase I			Phase II	
1	−0.0019	−0.0003	21	−0.0652	−0.0460
2	−0.0065	−0.0024	22	−0.0822	−0.1449
3	−0.0779	−0.0170	23	−0.3066	−0.3486
4	−0.0728	−0.0152	24	−0.3201	−0.5074
5	−0.0541	−0.0126	25	−0.2751	−0.6467
6	−0.0683	−0.0144	26	−0.2611	−0.7961
7	−0.0713	−0.0151	27	−0.5006	−1.0739
8	−0.0722	−0.0155	28	−0.4378	−1.1885
9	−0.0705	−0.0154	29	−0.3982	−1.2004
10	−0.0750	−0.0165	30	−0.4991	−1.3849
11	−0.0712	−0.0162	31	−0.4739	−1.4190
12	−0.0511	−0.0148	32	−0.6000	−1.6047
13	−0.0641	−0.0151	33	−0.7216	−1.7792
14	−0.0689	−0.0155	34	−0.7802	−1.9073
15	−0.0658	−0.0154	35	−0.8428	−2.0280
16	−0.0592	−0.0154	36	−0.8733	−2.1204
17	−0.0742	−0.0181	37	−0.8396	−2.1664
18	−0.0515	−0.0175	38	−0.7804	−2.1903
19	−0.0413	−0.0163	39	−1.0337	−2.3189
20	−0.0543	−0.0163	40	−0.7368	−2.2091

## Illustrative Example

In this section, an illustrative example has been presented based on some hypothetical dataset. The respective hypothetical study has been carried out by generating the variable of interest followed by the section "Proposed control chart" steps.

**Figure 9:**
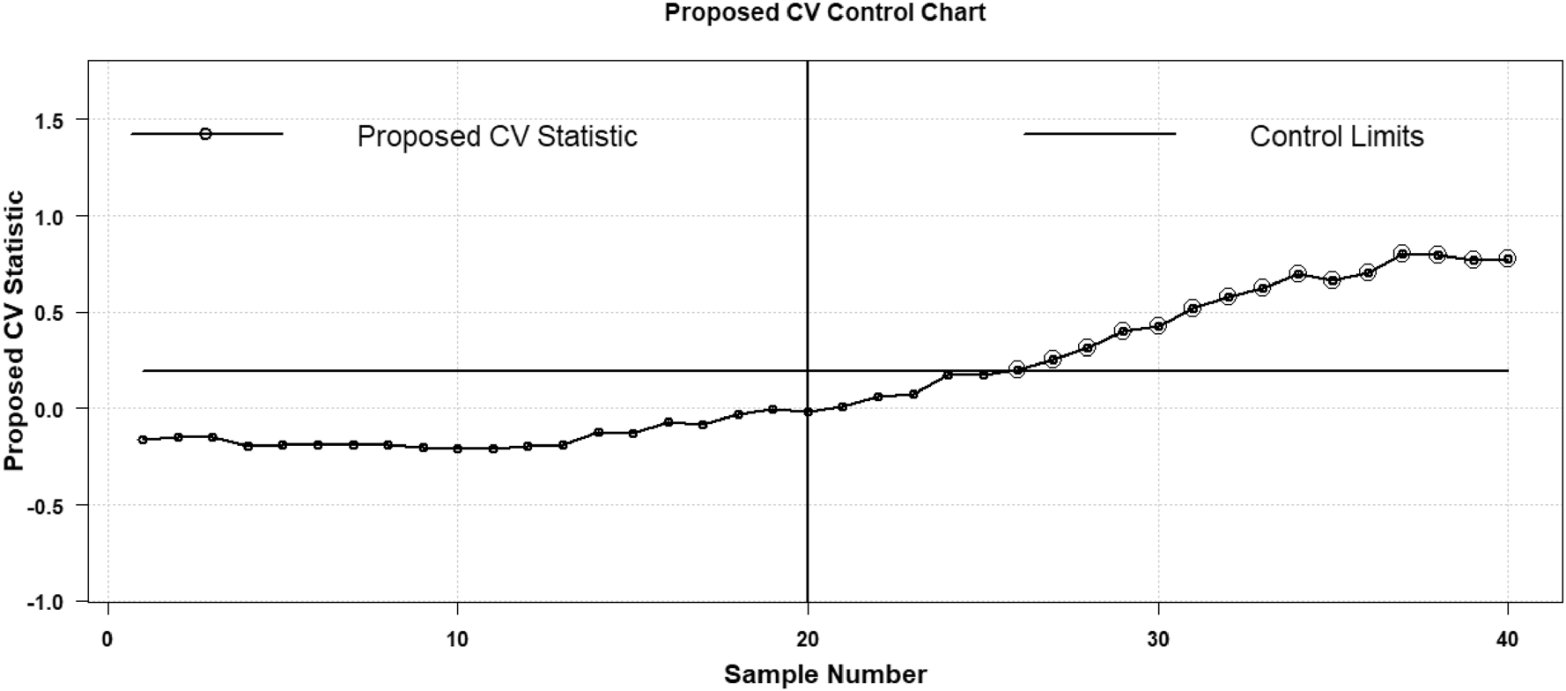
Proposed AAEWMA CV control chart (Illustrative Example).

**Figure 10:**
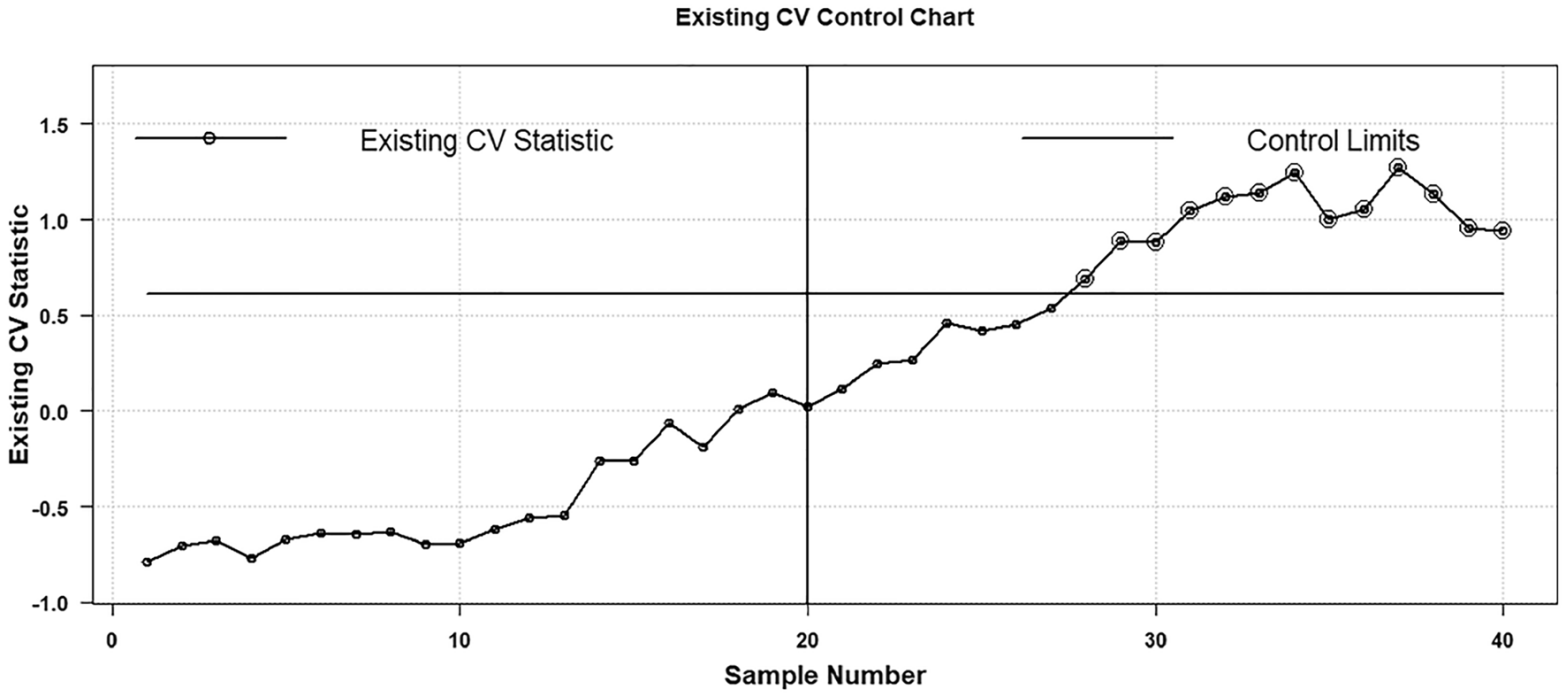
Existing AEWMA CV control chart (Illustrative Example).

Thus, the presented hypothetical data-based example has been employed with a one-sided decreased CV shit. To exhibit the comparison two control charts are constructed the proposed AAEWMA CV and the existing AEWMA CV control charts. Two respective phases are shown to exhibit the impact of shifts in phase II as the process incorporated with a decreased shift ${\varvec{\delta}}=0.5$ in process CV. Phase I is regarded as the in-control phase with no process CV shift to be taken as ${\varvec{\delta}}=1.0$. Over there is $m=40$ number of iterations, amongst them Phase I contains first in control $m=20$ samples each of size $n=5$ are drawn and the CV is computed followed by the design methodology mentioned in section "Proposed control chart". The in-control threshold is ${ARL}_{0}\cong 370$ with the value of $\mathfrak{h}=0.1946$ for the proposed and $\mathfrak{h}=0.6125$ for the existing Haq and Khoo^27^ for $\psi =0.1$. The values of ${\widehat{\gamma }}_{i}$ CV is determined to examine the impact of the decrease shift as ${\gamma }_{1.}$ The impact of increased shift is already discussed with a real dataset example. So, the two respective control charts are shown in Figs. 9 and 10. Both the plotting statistics ${\mathcal{A}}_{t}$ and ${H}_{t}$ computed values are plotted separately that exhibit the out-of-control trend in a more obvious manner. From the charts, it is clear that the existing AEWMA CV chart provides detection at the 28^th^ sample point, while the proposed AAEWMA CV chart detects at the sample point 26^th^. Therefore, it is suggested in favor of the proposed chart that is more effective than its existing chart methodology.

## Limitations

The CV monitoring AEWMA control chart is suggested in this manuscript, under a limitation for identifying the changes in both the mean and the variance, that variance is taken as the linear function of the mean. The utilization of the AEWMA chart design to monitor process CV, no doubt enhances the efficacy of the existing CV control chart.

However, there are limitations to using a CV control chart:

### Assumption of constant ratio

This limitation makes this control chart useful assuming that mean and standard deviation both move in a constant ratio concerning each other. The major impact of this limitation made monitoring through a CV control chart efficient because the CV chart can detect shifts in variations more effectively even when the process mean is not operating with a constant impact over time. The CV control chart assumes that the ratio of standard deviation to mean remains constant over time. If this assumption is violated, the chart may produce false signals or fail to detect shifts accurately.

### Sensitivity to outliers

CV is sensitive to extreme values in the data, which can distort its calculation and affect the interpretation of the control chart. Outliers may need to be treated or analyzed separately to ensure the integrity of the monitoring process. These outliers are the reason for the shift in the variation and are well-treated as well identified through a CV control chart.

### Interpretation challenges

Interpreting changes in CV values may be more challenging compared to traditional control charts. Understanding the underlying process dynamics and the relationship between mean and variance is crucial for accurate interpretation. But once it is overcome, well explained, and decided to be specific for the use of a CV control chart then it is far more effective than any other design of control chart.

### Limited applicability

The CV control chart is most effective when the process variance is directly linearly related to the mean. In situations where this relationship does not hold, alternative control charting methods may be more appropriate. This limitation is obvious, clear, and specific for the implementation of the CV control chart design to monitor a production process.

In summary, while the CV control chart provides a valuable tool for monitoring shifts in both mean and variance, its effectiveness relies on the assumption of a constant ratio between standard deviation and mean. Careful consideration of its limitations and appropriate interpretation of results enhances the efficacy of the suggested adaptive CV EWMA control chart exclusively. The determined results proved the strength of the argument that in the presence of a linear relation between the mean and the SD the utility of the CV chart with an adapted statistic, is essential for effective monitoring design application in quality control and process improvement initiatives.

## Future recommendations

For future recommendations regarding the use of CV control charts and their improvement, consider the following:

**Integration with other control chart designs:** Instead of relying solely on a CV to construct a control chart, consider integrating AEWMA statistic which is suggested in this manuscript with other designs such as combining the multiple control charts, and the use of other sampling schemes can provide a more comprehensive understanding of process variation and enhance the detection of process shifts and anomalies.

**Dynamic control limits:** Implement dynamic control limits that adjust based on the historical performance of the process. Dynamic control limits can better accommodate shifts in the process mean and variability over time, leading to improved sensitivity and reduced false alarms. The adaptation of the parametric settings as per process shift on a real-time basis will enhance the concept efficacy.

**Non-parametric approaches:** Explore non-parametric approaches for monitoring process variability, especially in cases where the underlying data distribution is unknown or non-normal. Non-parametric AEWMA control charts, such as the Median Absolute Deviation (MAD) AEWMA control chart, offer alternatives to CV control charts and can be more robust to deviations from normality.

## Conclusion

A novel adaptive CV EWMA control chart is introduced in this work, named the ‘AAEWMA CV control chart’ using the function given by Sarwar and Noor-ul-Amin^29^. If the process mean and SD have a consistent and proportionate relationship then a CV monitoring chart is appropriate to use such a manufacturing unit. Since fragile production units come from some sensitive manufacturing environments this proportionality component is inevitably situation-based in real-life scenarios. To handle these situations more effectively the mean and the dispersion monitoring charts alone are not sufficient. Therefore, to effectively detect changes in the process CV, it is proved in this study that is being presented that CV be monitored utilizing the adaptive design. Here, the strategy to adapt the value of the SCby Sarwar and Noor-ul-Amin^29^ is given on real-time data and is used to design the proposed AAEWMA CV control chart. The suggested chart is supposed to be implemented using a univariate dataset and the parent distribution is the normal distribution. The estimated SC value is derived from the estimation of the real-time basis sample data and is a variant for the given range. Therefore, although the process shift size is unknown ahead of time, it can be anticipated to fall within a given range due to the research of phase I information before the implementation of the appropriate control chart.

As a result, the proposed AAEWMA CV control chart design rapidly notices even slight changes in the process CV as shifts. In this strategy, the plotting statistic is adjusted based on the shift size and it is computed using this estimated SC value. The precise and efficient shift detection ability of the proposed AAEWMA CV control chart is studied extensively by using MC simulations and determined results are comprehensively discussed through tabular analysis for the various parametric settings. the comparative analysis reveals that the proposed AAEWMA CV control chart moves with the smaller RL value trend and indicates that the proposed AAEWMA CV control chart has a substantial performance than its counterparts. Two examples based on a real dataset and hypothetical data are provided to further explain how the chart can be effectively applied to the counterpart. both the example sections are extensively explained and elaborated with figures respectively to explain the point of effectiveness. The limitations and further research prospects are also discussed in separate sections.

## Data Availability

The datasets used and/or analyzed during the current study are available from the corresponding author upon reasonable request.

## References

[1] Mondal PP, Galodha A, Verma VK (2023). Review on machine learning-based bioprocess optimization, monitoring, and control systems. Biores. Technol..

[2] Shewhart WA (1926). Quality control charts. Bell Syst. Tech. J..

[3] Roberts S (1959). Control chart tests based on geometric moving averages. Technometrics.

[4] Page ES (1954). Continuous inspection schemes. Biometrika.

[5] Sanusi RA, Teh SY, Khoo MB (2020). Simultaneous monitoring of magnitude and time-between-events data with a Max-EWMA control chart. Comput. Ind. Eng..

[6] Alevizakos V, Chatterjee K, Koukouvinos C (2021). The triple exponentially weighted moving average control chart. Qual. Technol. Quant. Manag..

[7] Noor-ul-Amin M, Javaid A, Hanif M, Dogu E (2022). Performance of maximum EWMA control chart in the presence of measurement error using auxiliary information. Commun. Stat.-Simul. Comput..

[8] Alduais FS, Khan Z (2023). EWMA control chart for Rayleigh process with engineering applications. IEEE Access..

[9] Kang CW, Lee MS, Seong YJ, Hawkins DM (2007). A control chart for the coefficient of variation. J. Qual. Technol..

[10] Castagliola P, Achouri A, Taleb H, Celano G, Psarakis S (2015). Monitoring the coefficient of variation using a variable sample size control chart. Int. J. Adv. Manuf. Technol..

[11] Abbasi SA (2020). Efficient control charts for monitoring process CV using auxiliary information. IEEE Access..

[12] Noor-ul-Amin M, Riaz A (2020). EWMA control chart for coefficient of variation using log-normal transformation under ranked set sampling. Iran. J. Sci. Technol. Trans. A: Sci..

[13] Tran PH, Heuchenne C, Nguyen HD, Marie H (2021). Monitoring coefficient of variation using one-sided run rules control charts in the presence of measurement errors. J. Appl. Stat..

[14] Shewhart, W. A. *Economic Control of Quality of Manufactured Product* (ASQ Quality Press, 1931).

[15] Hong E-P, Kang C-W, Baek J-W, Kang H-W (2008). Development of CV control chart using EWMA technique. J. Soc. Korea Ind. Syst. Eng..

[16] Castagliola P, Celano G, Psarakis S (2011). Monitoring the coefficient of variation using EWMA charts. J. Qual. Technol..

[17] Zhang J, Li Z, Chen B, Wang Z (2014). A new exponentially weighted moving average control chart for monitoring the coefficient of variation. Comput. Ind. Eng..

[18] Calzada ME, Scariano SM (2013). A synthetic control chart for the coefficient of variation. J. Stat. Comput. Simul..

[19] Castagliola P, Achouri A, Taleb H, Celano G, Psarakis S (2013). Monitoring the coefficient of variation using control charts with run rules. Qual. Technol. Quant. Manag..

[20] Yeong WC, Khoo MBC, Lim SL, Teoh WL (2017). The coefficient of variation chart with measurement error. Qual. Technol. Quant. Manag..

[21] Teoh WL, Khoo MB, Castagliola P, Yeong WC, Teh SY (2017). Run-sum control charts for monitoring the coefficient of variation. Eur. J. Oper. Res..

[22] Muhammad ANB, Yeong WC, Chong ZL, Lim SL, Khoo MBC (2018). Monitoring the coefficient of variation using a variable sample size EWMA chart. Comput. Ind. Eng..

[23] Yeong WC, Lim SL, Khoo MBC, Castagliola P (2018). Monitoring the coefficient of variation using a variable parameters chart. Qual. Eng..

[24] Noor-ul-Amin M, Tariq S, Hanif M (2019). Control charts for simultaneously monitoring of process mean and coefficient of variation with and without auxiliary information. Qual. Reliabil. Eng. Int..

[25] Riaz, A., & Noor-ul-Amin, M. Improved simultaneous monitoring of mean and coefficient of variation under ranked set sampling schemes. In *Communications in Statistics-Simulation Computational Statistics* 1–17 (2020).

[26] Capizzi G, Masarotto G (2003). An adaptive exponentially weighted moving average control chart. Technometrics.

[27] Haq A, Khoo MB (2019). New adaptive EWMA control charts for monitoring univariate and multivariate coefficient of variation. Comput. Ind. Eng..

[28] Noor-ul-Amin M, Noor S (2020). An adaptive EWMA control chart for monitoring the process mean in Bayesian theory under different loss functions. Qual. Reliabil. Eng. Int..

[29] Sarwar MA, Noor-ul-Amin M (2022). Design of a new adaptive EWMA control chart. Qual. Reliab. Eng. Int..

[30] Arshad A, Noor-ul-Amin M, Hanif M (2021). Function-based adaptive exponentially weighted moving average dispersion control chart. Qual. Reliabil. Eng. Int..

[31] Noor-ul-Amin M, Arshad A, Hanif M (2021). A function based adaptive EWMA mean monitoring control chart. Quality and Reliability Engineering International..

[32] Montgomery D. Introduction to Statistical Quality Control, Sixth Edition. In *Arizona* (2009).

[33] Jiang W, Shu L, Apley DW (2008). Adaptive CUSUM procedures with EWMA-based shift estimators. IIE Trans..

